# Digital Behavioral Activation Interventions During the Perinatal Period: Scoping Review

**DOI:** 10.2196/40937

**Published:** 2023-02-28

**Authors:** Elisa Mancinelli, Gaia Dell'Arciprete, Davide Pattarozzi, Silvia Gabrielli, Silvia Salcuni

**Affiliations:** 1 Department of Developmental and Socialization Psychology, University of Padova Padova Italy; 2 Digital Health Lab, Centre for Digital Health and Wellbeing, Fondazione Bruno Kessler Trento Italy

**Keywords:** behavioral activation, eHealth, perinatal care, depression symptoms, scoping review, mobile phone

## Abstract

**Background:**

Pregnancy is a complex period that implies many biopsychosocial changes, and the way women adapt to these changes impacts their well-being and the chances of developing mental health problems. During the perinatal period, women have expressed a preference for support delivered on the web. In this regard, interventions such as behavioral activation (BA), which are brief and structured psychosocial interventions, seem particularly suited to be delivered through digital solutions.

**Objective:**

This study aimed to map the literature investigating digital BA interventions deployed during the perinatal period. We paid particular attention to the methodological underpinnings of the studies, the potential impact of BA interventions on symptoms other than depression, and the existence of differences occurring when these interventions were administered during pregnancy versus the postpartum period.

**Methods:**

A systematic search compliant with the PRISMA-ScR (Preferred Reporting Items for Systematic Reviews and Meta-Analysis extension for Scoping Reviews) guidelines was conducted considering 5 bibliographic databases; reference lists and key journals were also screened by 2 independent authors following a double-blind approach.

**Results:**

A total of 7 studies published between 2013 and 2022 were included. In total, 2 studies were protocols for randomized controlled trials, 5 were empirical studies, and 1 was a qualitative study. All studies focused on the postpartum period, except for 1 that focused on the broader perinatal period. Promising effects on depression symptoms were reported but not on other psychosocial symptoms. Low intervention adherence has emerged, whereas the usability associated with the digital means used to deploy interventions was scarcely addressed; moreover, information on the digital platforms used was poorly reported overall.

**Conclusions:**

Our findings highlight the scarcity and preliminary nature of digital BA interventions deployed during the perinatal period, where the focus seems more on treatment rather than prevention. Moreover, future studies should also consider and address usability and user engagement, given their relevance to intervention efficacy.

## Introduction

### Background

The transition to motherhood is a life-changing experience entailing a series of social, psychological, and hormonal changes, which may be challenging to adapt to and often cause exhaustion, a sense of overwhelm, and fatigue [1,2], thereby directly impacting pregnant and postpartum women’s quality of life and overall well-being. Taken together, these factors define the perinatal period as a high-risk period for women’s mental health [3,4]. The literature provides plenty of evidence on this matter, highlighting how pregnancy is often associated with mood instability [5] and common mental disorders (1%-37%) [6] as well as depression (approximately 25.3%) [6,7] and anxiety (1%-26%) [6]. Depression symptoms during pregnancy, in particular, are among the main predictors of postpartum depression [8]. From a clinical viewpoint, depression during the perinatal period is described as peripartum depression, which consists of an episode of major depression with peripartum onset, satisfying the criteria for either major depression or persistent depressive disorder [9]. The direct association of peripartum depression with the peculiar challenges and bodily changes intrinsic to the perinatal period makes the condition peculiar, thereby determining the need to consider it as a stand-alone disorder [10]. Accordingly, a recent review concluded that peripartum depression “may be distinct from major depressive disorder with respect to symptom severity, hormone contributions, heritability, epigenetic mechanisms, and response to standard and novel treatment interventions” [10].

Considering the detrimental effects that depression symptoms, together with the often associated anxiety and stress symptoms, have on the physical and psychological well-being of both mothers and children [4], it is paramount to take prompt action and minimize the incidence of adverse effects. In this regard, it is important to emphasize that in the present age, beside the abovementioned challenges, the perinatal period can be experienced very differently by women. Physiological pregnancies and artificially induced ones, that is, pregnancies reached through assisted reproductive techniques (ARTs; eg, in vitro fertilization and intracellular sperm injection), can particularly be distinguished between in this regard. As a matter of fact, ARTs are infertility treatment that determine a series of specific challenges that differentiate them from physiological pregnancies, such as increased psychological distress, loss of self-esteem, relationship problems, disruption in personal life, and even economic problems linked to the substantial expenses they entail [11-14]; this determines a greater psychological toll compared with physiological pregnancies. Moreover, ART are associated with an increased risk of miscarriage [15,16], which could further hinder women’s adaptation capacities; although having reached pregnancy, many women might still be mourning their previous interrupted ones [17]. For these reasons, physiological pregnancies, ART-induced pregnancies, and consequent postpartum periods must be deemed as very different experiences. Accordingly, it is paramount to account for their peculiarities in clinical practice by tailoring interventions specifically to their respective needs.

In this regard, contrary to women undergoing ART who are kept under greater clinical scrutiny by default, access to mental health care for women at large going through the perinatal period is severely limited by a series of logistic challenges; for instance, time constraints, lack of information about services, and social stigma are indicated by women themselves as the main obstacles hindering their ability and willingness to seek help [18-20]. However, such barriers can be overcome through the implementation of digital interventions; the literature not only highlights a clear preference for support delivered through a web-based format expressed by women experiencing mental health issues during the postpartum period [21-23] but also underscores how such treatments are effective in decreasing the severity of mood disorders, both during pregnancy and the postpartum period [24-26].

In this regard, behavioral activation (BA) interventions, which in their modern protocols can be counted among the so-called “third wave” cognitive and behavioral therapies [27], seem particularly suited to be implemented digitally. BA is a brief, structured, and empirically supported psychotherapeutic approach developed as a stand-alone treatment for depression [28]. Laying its foundation on the behavioral model, it is mainly aimed at increasing engagement in adaptive and pleasurable activities and decreasing engagement in maladaptive behaviors through the systematic targeting of patients’ escape and avoidance strategies [29,30]. The focus is exclusively on the promotion of behavioral change, stimulated by using a series of different strategies such as self-monitoring, activity scheduling, values and goals assessment, and skills training. The ultimate goal is to encourage patients to act in line with their values, allowing them to reconnect with sources of positive reinforcement and fostering a sense of well-being, agency, and mastery through the reconstruction of a routine [30]. Both the efficacy and effectiveness of in-person BA as a treatment specific for depression have been widely proven [31], and the literature also provides promising evidence regarding its efficacy in perinatal depression, both in terms of improved outcomes [29,32] and of engagement and satisfaction [33]. The distinctive features of BA render it a parsimonious, transportable, simple to implement, and cost-effective treatment, suitable to be administered by both specialists and generic mental health professionals [34] in a wide range of formats such as web-based interventions or self-guided smartphone apps [29]. Encouraging findings already exist on the feasibility of internet-based BA interventions for adults with symptoms of depression [35]; therefore, it seems appropriate to further investigate the topic within the context of perinatal care. Nonetheless, it is worth mentioning that evidence on the efficacy and specific features of BA interventions during the perinatal period is still scarce and inconspicuous. For instance, there is some initial evidence highlighting that the effect of in-person BA on depression symptoms might also generalize to other symptoms, thereby leading to the simultaneous reduction of the highly associated anxiety and stress [29,30,32]. However, studies investigating the efficacy of BA interventions have classically focused on the mere reduction of depression symptoms; thus, further research is needed to deepen our knowledge and provide solid evidence on its effects on other concurrent mental health conditions. Similarly, although as previously mentioned, existing studies regarding digital interventions show promising results, and it must be noted that it is a fairly new field and is still under development; therefore, further studies are needed in this area as well [35,36]. Accordingly, this scoping review aimed to map the available literature on digital BA interventions administered specifically during the perinatal period. Scoping reviews serve to bring together “literature in disciplines with emerging evidence, as they are suited to addressing questions beyond those related to the effectiveness or experience of an intervention” [37]. Indeed, this methodological approach to literature review allows us to map the range, nature, and extent of existing research evidence on a given topic regardless of the specific study design while also allowing the identification of gaps within the available literature [38]. Accordingly, this scoping review intended to investigate how BA interventions have been deployed through digital solutions, paying particular attention to their methodological underpinnings; a further aim was to assess if studies have considered the potential of digital BA interventions to influence symptoms and conditions other than depression (eg, anxiety and stress symptoms, overall quality of life, and well-being). This was expected to provide insights useful for refining the existing digital BA interventions. Moreover, it was also expected to support the further development of effective prevention and intervention programs divulged through digital solutions for women in the perinatal period, thereby supporting both their own and their children’s well-being.

### Objectives

The specific research questions guiding this scoping review were as follows: (1) How have BA interventions been structured (eg, intervention length, number of modules, topics considered, and guided vs unguided interventions) to be administered through digital means? (2) Has a specific BA protocol been followed? If so, on what BA protocol were the interventions based? [39] (3) What were the main barriers, including both participants’ concerns and issues given by the digital tool itself, in implementing BA interventions through digital means? (4) Were digital BA interventions able to influence psychosocial symptoms other than depression? (5) Have they willingly been used to influence symptoms other than depression? and (6) Are there differences in the BA interventions administered during the antenatal period versus the postnatal period?

## Methods

This scoping review was conducted in compliance with the PRISMA-ScR (Preferred Reporting Items for Systematic Reviews and Meta-Analysis extension for Scoping Reviews) guidelines (refer to Table S1 in Multimedia Appendix 1 [40-47]).

### Eligibility Criteria

The inclusion criteria for this study were as follows: (1) considering BA interventions administered through digital means (web-based, smartphone-based, telehealth, etc), (2) inclusion of women aged ≥18 years, (3) focusing on the peripartum period (both antenatal and post partum), (4) inclusion of women who were experiencing or had experienced a physiological pregnancy (up to 1 year post partum), and (5) being written in English.

The exclusion criteria were as follows: (1) being a review article, (2) focusing on or including women who were experiencing or had experienced artificially induced pregnancy, and (3) focusing on or including women with preexisting medical conditions.

In line with the intent of scoping reviews, that is, mapping the available literature regardless of its specific study design [38], no restrictions were imposed on the study design. Accordingly, any type of study satisfying the criteria mentioned above was included, as the intent was to provide an overview of existing literature that is informative of what has already been done (eg, already published studies), what is expected to be done in the near future (eg, study protocols), and what is still lacking entirely or needs further investigation (ie, literature gaps). For the same reason, studies relying on secondary data were also included if they provide information that adds up to the primary analysis, thereby allowing a more in-depth understanding of the intervention evaluated and, more broadly, of the state of the art.

### Search Strategy

A total of 5 electronic bibliographic databases, namely, Web of Science, PubMed, PsycINFO, Embase, and CINAHL, were screened in April 2022 to identify studies that met the eligibility criteria. No search restrictions were applied. Reference lists were also scanned, and a handsearching of key journals (ie, *Journal of Medical Internet Research* and *Telemedicine and e-Health*) was performed to ensure a comprehensive literature search. Studies were identified using the following search strings: (behavioral activation OR behavioral activation OR activity scheduling OR pleasant events OR pleasant event) AND (digital interventions OR telehealth OR telemedicine) AND (perinatal depression OR antenatal depression OR postpartum depression OR perinatal mental health); (behavioral activation) AND (web-based interventions OR e-health OR internet-based interventions) AND (perinatal OR postnatal OR prenatal OR antenatal OR postpartum OR maternal OR pregnant OR pregnancy).

### Study Screening and Data Charting Process

A double-blind screening of titles and abstracts was performed using the ASReview Lab software [48] by 2 authors (EM and DP), who also manually inspected the software results and then performed the subsequent full-text screening in a double-blind fashion. Any doubts or disagreements were resolved by consulting a third author (SS). Similarly, 2 independent authors (EM and DP) performed the data extraction in a double-blind fashion. Any disagreement was resolved by consulting a third author (SG). The extracted data, collected in an Excel (Microsoft Corporation) sheet, were as follows: *study characteristics* (digital object identifier; first author’s name; publication year; country of origin; study design; study aim; time points for data collection; outcomes considered by the included study; measurement tools used to evaluate the outcomes; main results; reason for dropout, if applicable; participants’ feedback on the intervention, if present; and type of comparator, if present), *sample characteristics* (sample size, age, ethnicity, occupation, marital status, educational level, income, clinical characteristics, gestational week or postpartum period, and inclusion and exclusion criteria for the participants), and *BA intervention characteristics* (description of the BA protocol, original BA protocol from which the new one was derived, if and how the protocol was modified, intervention length and structure, digital means used to administer the BA, and intervention delivery format).

## Results

### Search Results

**Figure 1:**
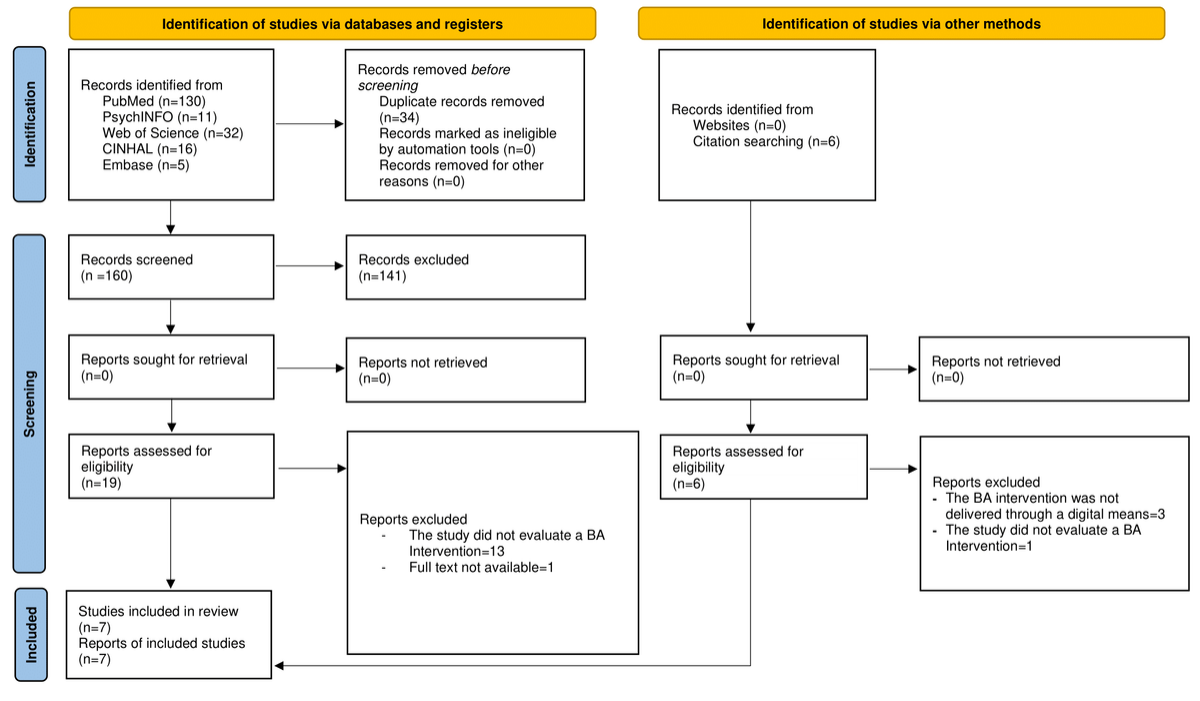
PRISMA (Preferred Reporting Items for Systematic Reviews and Meta-Analyses) 2020 flow diagram, including searches of databases, registers, and other sources. BA: behavioral activation.

The database search yielded 194 studies (Figure 1). Following duplicate removal, the titles and abstracts of 160 studies were screened; 141 studies were removed, resulting in 19 studies whose full text was screened in accordance with the inclusion and exclusion criteria. Of these 19 studies, 5 (26%) were eventually included. The database search was integrated with a manual search of key journals and the screening of reference lists, through which 7 more studies were identified for full-text screening; only 2 studies complied with the inclusion criteria. Taken together, 7 studies were ultimately included in this scoping review. Excluded studies with reasons for exclusion are reported in Tables S2, S3, and S4 in Multimedia Appendix 1.

### Studies’ Characteristics

**Figure 2:**
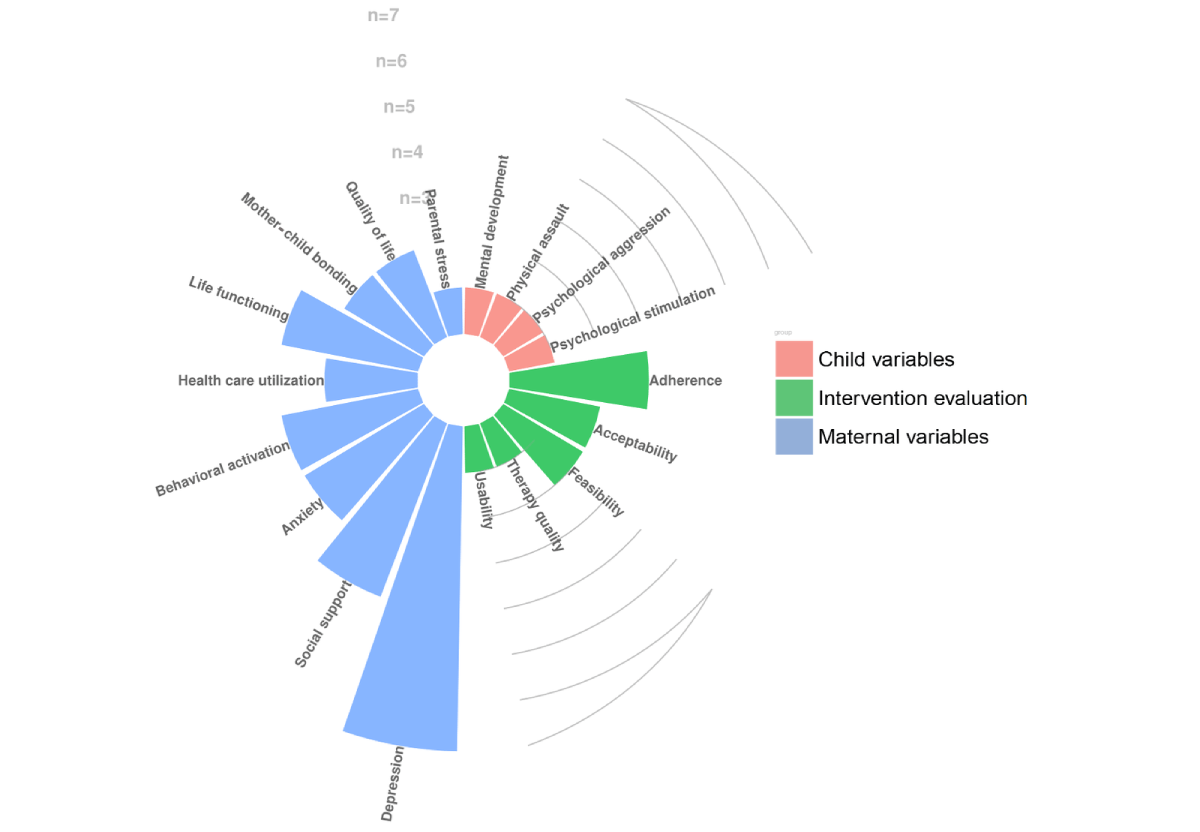
Circular bar plot of the main outcomes of the studies. n=frequency of the outcomes reported by the included studies.

The aims and design of the studies are reported in Table 1, and the studies’ main outcomes are summarized in Figure 2. The included studies were published between 2013 and 2022, of which 2 are randomized controlled trials (RCTs) [41,42] and 2 are protocols for RCTs [43,44]. Moreover, 2 studies are secondary analyses conducted on the RCT by O’Mahen et al [42], 1 is a secondary analysis focused on a subsample of the original sample [45], and 1 is a secondary analysis focused only on follow-up data of the total sample [46]. Finally, 1 is a qualitative study, which included participant-level analyses as well as 2 case studies [47]. Both the studies by Singla et al [44,47] relate to the same broad ongoing study. Only the 2 protocol studies [43,44] preregistered their clinical trials. All included studies were focused on evaluating the efficacy or effects of a digital BA intervention, except for 1 study [45], which investigated the processes underlying digital BA’s efficacy. The outcomes considered by the included studies are summarized in Figure 2. Although all interventions were developed to target depression symptoms, coherently with the intents of BA, studies have also marginally considered other maternal variables such as anxiety symptoms (n=3) [43,44,46], parental stress (n=1), quality of life (n=1) [43], social support (n=4) [42,44,46], work and social functioning (n=3) [42,45,46], and mother-child bonding (n=3) [42-44]. Moreover, only Obikane et al [43] and Singla et al [44] included child-related variables in their protocol study, such as maternal psychological and physical aggression toward the child (n=1) [43], the child’s physiological (n=1) [43] or mental development (n=1) [44], and the level of health care services used for the child (n=2) [43,44] (Figure 2; Table S5 in Multimedia Appendix 1). The included studies also marginally evaluated the intervention-related outcomes. Specifically, 1 study protocol [44] reported that they will evaluate therapy quality as well as homework completion and the frequency with which the intervention sessions are followed. Instead, 3 studies [44-46] evaluated intervention adherence, operationalized as the number of intervention modules opened and completed [45,46]. Only 2 studies specifically considered interventions’ feasibility (ie, if the intervention is feasible for further testing) and acceptability (ie, how well the intervention meets the needs of the target population) [41,43]. Finally, only 1 study [43], a study protocol, reported that the assessment of usability will be conducted through the System Usability Scale [49].

**Table 1:** Aims and characteristics of the studies.

Study	Study type	Study aim
Bagnall [46], 2014	Long-term follow-up of RCT^a^ (doctoral dissertation)	To collect 16-month follow-up data of postnatal women who participated in a feasibility RCT investigating an internet-based BA^b^ [42] To investigate intervention efficacy at a 16-month follow-up To investigate the intervention adherence and predictors of intervention outcomes To investigate the predictive role of psychological or demographic factors of attrition throughout the study and at follow-up
Obikane et al [43], 2021	Protocol for RCT	To investigate the efficacy of a postnatal internet-based BA program targeting postpartum depression in reducing depression symptoms (primary aim) as well as mother-child bonding, parental stress, and quality of life To investigate the program’s efficacy in preventing child psychological and physical abuse and to evaluating the associated child developmental measures To investigate the program’s acceptability, feasibility, and appropriateness and their association with the participants’ medical and social characteristics
O’Mahen et al [41], 2014	RCT	To investigate the feasibility (primary aim), assessed as treatment adherence, of an internet-based BA program targeting postpartum depression To investigate the program’s efficacy in reducing depression symptoms compared with usual care
O’Mahen et al [42], 2014	RCT	To investigate the feasibility, assessed as treatment adherence, and to identify adherence predictors (primary aims) of an internet-based BA program targeting postpartum depression To investigate the program’s efficacy in reducing depression and anxiety symptoms as well as functional impairment, perceived support, and mother-child bonding
O’Mahen et al [45], 2017	Secondary analysis from the study by O’Mahen et al [42]	To investigate the participants’ processes referred to sudden gains and depression spikes associated with an internet-based BA program targeting postpartum depression [42] To investigate the association between participants’ sudden gains and therapists’ actions
Singla et al [44], 2021	Protocol for 4-arm RCT (noninferiority trial)	To investigate if a BA intervention targeting postpartum depression can be delivered by nonspecialists (primary aim) To compare the efficacy of a BA intervention in reducing depression symptoms delivered through telemedicine versus delivered in person (primary aim) To investigate the efficacy of the BA intervention in reducing anxiety symptoms To investigate the comparative effectiveness of the BA intervention delivered antenatally versus postnatally To investigate whether the timing of the BA intervention administration differently influences the child’s mental development To investigate barriers, facilitators, and processes subsuming the BA intervention efficacy in reducing depression and anxiety symptoms
Singla et al [47], 2022	Qualitative study	To qualitatively investigate the barriers and facilitators of BA intervention delivered through telemedicine from both participants’ and intervention providers’ perspectives (ongoing trial)

^a^RCT: randomized controlled trial.

^b^BA: behavioral activation.

### Sample Characteristics

The sample characteristics and studies’ sample-related inclusion criteria are summarized in Table 2. All participants were postpartum women, except for those in the study by Singla et al [44,47], which focused on both antenatal and postnatal women. All studies used the Edinburgh Postnatal Depression Scale to include women in their study, albeit relying on different cutoff values. Moreover, in their 2014 study, O’Mahen et al [42,45,46] had supplemented the Edinburgh Postnatal Depression Scale with a telephone interview aimed at ensuring that women satisfied the criteria for major depressive disorder according to the International Classification of Diseases, Tenth Revision. With regard to demographic information (Table S6 in Multimedia Appendix 1), information on the sample’s age was reported by only 2 studies [42,47], the sample’s ethnicity by 3 studies [42,44,45], occupation by 4 studies [41,42,45,47], educational level by 3 studies [41,42,45], income by 2 studies [42,45], marital status by 4 studies [41,42,45,47], and the total number of children by 3 studies [41,42,45]. Finally, with regard to the studies’ sample sizes, the 2 protocol studies included [43,44] stated that they will include samples whose numerosity will be based on preliminary power analyses, and all the other included studies reported small sample sizes. Only 1 study [41] reported a larger sample size (<150); nonetheless, changes in the sample’s numerosity from baseline to intervention end point and follow-up are unclear.

**Table 2:** Sample characteristics.

Study	Sample, N	Perinatal period	Inclusion criteria	Exclusion criteria
Obikane et al [43], 2021	Over 75 per group (4 groups: estimated)	Post partum	Aged ≥20 years (adult age in Japan) Having given birth within the past 10 weeks Available internet-access Living with the newborn baby Fluent in Japanese	Suicidal intent Receiving public livelihood assistance
O’Mahen et al [41], 2013	EG^a^: 164; CG^b^: 134	Post partum	Aged ≥18 years EPDS^c^ >12 Having given birth within the past 12 months Being a member of Netmums	Not reported
O’Mahen et al [42], 2014; Bagnall [46], 2014	EG: 41; CG: 42	Post partum	Aged ≥18 years Having given birth within the past 12 months EPDS >12 MDD^d^ (International Classification of Diseases, Tenth Revision criteria)	Substance abuse Psychosis
O’Mahen et al [45], 2017	32 (subsample of the study by O’Mahen et al [42])	Post partum	As per the study by O’Mahen et al [42]	As per the study by O’Mahen et al [42]
Singla et al [44], 2021	342 per group (4 groups; estimated)	Perinatal	Aged ≥18 years EPDS ≥10 Pregnant up to 36 weeks or 4-30 weeks post partum Speaks English or (US sites) Spanish	Suicidal intent Active symptoms of psychosis or mania Taking psychotropic medication Change of medication 2 weeks before enrollment Ongoing psychotherapy Substance abuse Severe fetal anomalies, stillbirth, or infant death for pregnant participants
Singla et al [47], 2022	23	Perinatal	As per the study by Singla et al [44]	As per the study by Singla et al [44]

^a^EG: experimental group.

^b^CG: control group.

^c^EPDS: Edinburgh Postnatal Depression Scale.

^d^MDD: major depression disorder.

### Digital Interventions’ Characteristics

The characteristics of the different interventions are reported in Table 3 and Table S7 in Multimedia Appendix 1. All interventions were based on validated BA protocols (ie, validated and manualized intervention protocols); however, none extensively explained the changes made to adapt the intervention to either the population or the digital setting. Studies’ intervention content structuring is quite consistent, with differences given mainly by the inclusion of optional modules [42,46] or the possibility to customize sessions [41]. Furthermore, in the qualitative study by Singla et al [47], the intervention protocol deployed during the COVID-19 lockdown was adapted to account for pandemic-related stressors and participants’ perceived racial injustice in a subsample of ethnic minority participants.

All interventions were guided, except for an unguided one [41] that, nonetheless, included a sort of “technical” support, whereby psychologists or specialized health visitors gave advice for homework completion and answered participants’ questions on the program material; no further information was reported. With regard to guided interventions, only O’Mahen et al [42] and Singla et al [44,47] reported information on how supporters guiding them had been trained and monitored through supervision by expert clinicians. Moreover, in the studies by Singla et al [44,47] and O’Mahen et al [42], authors monitored and evaluated supporters through independent fidelity raters who listened to recordings of the sessions. All 3 studies by O’Mahen et al [41,42,45] and the related secondary analysis by Bagnall [46] considered the web-based Netmums BA program; both the studies by Singla et al [44,47] relied on telemedicine, whereas only Obikane et al [43] reported the development of a web-based BA intervention that can be deployed through smartphones. Obikane et al [43] further reported that they will ensure data confidentiality by temporarily storing data in the Amazon Elastic Compute Cloud system and will only later move them in a password-protected computer. No other study reported information referred to data privacy and storage.

**Table 3:** Characteristics of the interventions.

Study	Intervention name	Length (weeks), total number of sessions	Guided versus unguided	Guided—description	Digital means	Intervention delivery format
Obikane et al [43], 2021	SmartMama	12, 12	Guided (psychotherapists)	Provided participants with feedback Answered participants’ questions about the intervention	Web-based app for smartphones	Not reported
O’Mahen et al [41], 2013	Postnatal iBA^a^	15, 11	Unguided	N/A^b^	Web based (Netmums site)	Multimedia presentations Web-based materials Web-based homework Possibility to access weekly web-based real-time “clinics” Web-based chat room moderated by parent supporters
O’Mahen et al [42], 2013; O’Mahen et al [45], 2014; Bagnall [46], 2014	NetmumsHWD	17, 12	Guided (mental health workers)	Answered participants’ questions about the intervention Support the overcoming of barriers to the implementation of the intervention	Web based (Netmums site)	Multimedia presentations Web-based materials Web-based homework Possibility to access weekly web-based real-time “clinics” Web-based chat room moderated by peer supporters
Singla et al [44], 2021	Behavioral activation	6-8, 6-8	Guided (nonspecialists and specialists)	Delivers the intervention Addresses treatment barriers and facilitators	Telemedicine	Zoom (Zoom Video Communications Inc.) or WebEx video calls
Singla et al [47], 2022	Behavioral activation	Not reported, 8	Guided (trained psychologists)	Delivered the intervention Addresses treatment barriers and facilitators Accounted for COVID-19 and racial injustice worries	Telemedicine	Zoom or WebEx video calls

^a^iBA: internet-based Behavioral Activation.

^b^N/A: not applicable.

### Results of Individual Evidence and Overall Synthesis

The main results of the studies, referring to both clinical outcomes and intervention-related outcomes, are summarized in Table 4. Overall, these studies consistently showed a significant intervention effect on depression symptoms. However, following multiple imputation analyses, the results of the study by O’Mahen et al [42] showed that such differences were not significant between the intervention and control groups. The same authors reported a trend for reduced anxiety symptoms and improved work and social functioning compared with the control condition at the intervention end point; however, this trend was not statistically significant. Accordingly, in the secondary follow-up analysis by Bagnall [46], the intervention effect was not significantly different between groups at any time point. Notwithstanding, the 2 case studies reported by Singla et al [47] were useful in highlighting the subjective experience of participants and their perceived benefits of the interventions. In both case studies, participants reported an improvement in communication skills that favored the support between them and their husbands by sharing child-related responsibilities. Moreover, at the end of the intervention, participants reported better associating their mood with what was happening in their lives, thereby voluntarily redirecting their attention toward value-based activities that would improve their mood. At the intervention end point, one of the women self-reported remission in both depression and anxiety symptoms, whereas the other reported that she was then able to embrace her past traumas, triggered by postpartum-related stressors, and thereby requested a referral to a clinical professional. The secondary analysis performed by O’Mahen et al [45] was also useful in highlighting the relevant processes underlying the intervention that were associated with its efficacy. Specifically, the authors observed that slightly more than half of the sample (18/32, 51%) showed at least one sudden improvement or gain in depression symptoms, even though these were often followed by a depression spike; however, participants showing these sudden improvements reported much reduced depression symptoms at intervention end point and also reported having followed more web-based intervention modules compared with those who had not reported such improvements. The same participants had also talked about more specific and concrete topics during the telephone sessions preceding the sudden gains [45].

Regarding the interventions’ feasibility, acceptability, and overall intervention adherence, the available information is limited. However, although studies explicitly evaluated these factors, their results on dropout rates and adherence seemingly suggest reduced acceptability. The studies by O’Mahen et al [41,42] provided information in this regard, reporting that dropouts were quite high and that there was limited participation in the real-time “online clinics” as well as in the chat rooms made available for mums to talk with each other. Moreover, limited intervention adherence was highlighted by the modest median completion rate of the intervention sessions. In this regard, it is noteworthy that reduced adherence was associated with women’s lower socioeconomic level, reduced social support as well as reduced work and social functioning, and with attending school or working [42]. Nonetheless, based on participants’ feedback, what had favored acceptability and adherence was the convenient delivery of the intervention’s content and the ease experienced in following the program; however, the latter is hindered when interventions include large number of sessions and activities that women are requested to follow [41]. Accordingly, O’Mahen et al [41,42] had simplified their intervention from their 2013 versus 2014 study. Moreover, contrary to what was reported by O’Mahen et al [42] regarding the predictors of intervention adherence, the study by Bagnall [46] did not identify any significant predictors (ie, depression symptoms level, level of BA, social support, and household income) of intervention adherence or the predictive role of assessments’ adherence to the intervention outcomes (eg, depression symptoms). The latter study was primarily focused on investigating participants’ adherence to follow-up assessments (which might thus better fit within the broader construct of “intervention feasibility” more than within that of “adherence”). In this regard, they reported that outreach helped improve participation in the follow-up assessments. No study investigated the programs’ usability; however, 1 of the included study protocols [43], as previously reported, is expected to provide further information in this regard.

**Table 4:** Main results of the studies.

Study	Assessment time points	Clinical outputs	Intervention-related outputs
Bagnall [46], 2014	T0^a^: baseline T1^b^: intervention end point T2^c^: follow-up, 10 months from intervention end point T3^d^: follow-up, 16 months from intervention end point	No significant reduction in depression and anxiety symptoms or improvements in work and social functioning as well as the level of BA^e^ emerged, at any time point, among participants from the intervention group compared with participants in the usual care condition	Intervention adherence was not associated with depression symptoms at any time point following the intervention Household income, relationship status, depression symptoms, and level of BA and social support at baseline were predictive of attrition
Obikane et al [43], 2021	T0: baseline T1: intervention end point T2: follow-up, 12 weeks from intervention end point	N/A^f^	N/A
O’Mahen et al [41], 2013	T0: baseline T1: intervention end point	Significant reduction in depression symptoms among participants from the intervention group compared with participants in the usual care condition	Appreciation for the program’s flexibility and convenient delivery Difficulty in keeping up with the program requirements Low participation in the chat rooms with other mothers and in the web-based clinics Drop in the sessions’ adherence following the second session
O’Mahen et al [42], 2014	T0: baseline T1: intervention end point T2: follow-up, 6 months from intervention end point	Trend of greater reduction in depression and anxiety symptoms and improvement in life function in the intervention group compared with participants in the usual care condition Significant greater chances of clinically improved depression symptoms (odds ratio 0.26, 95% CI 0.10-0.71) in the intervention group compared with the control condition	Modest intervention adherence overall Reduced intervention adherence among women (1) working or attending school, (2) with less social support, (3) reduced life functioning at baseline, and (4) with a lower socioeconomic status Only 5% completed ≥8 sessions, of which 5 completed all 12 sessions Most chosen optional module was the one on motherhood (chosen by 22% of participants)
O’Mahen et al [45], 2017	As per the study by O’Mahen et al [42]	Overall fast initial improvement in depression symptoms (particularly among women with higher baseline symptoms), which slowed down over time In total, 51% of the sample had a sudden gain (ie, clinically relevant improvement in depression symptoms); associated with reduced depression symptoms overall at the intervention end point In total, 19% of the sample showed depression spike In total, 75% of the sample that had experienced a depression spike and also showed a sudden gain, with the latter preceding the depression spike Women showing more sudden gains had completed more web-based modules but not more telephone sessions	N/A
Singla et al [44], 2021	T0: baseline T1: 3 months from randomization T3: 6 months from randomization T4^g^: 12 months from randomization	N/A	N/A
Singla et al [47], 2022	N/A	The 2 case studies highlighted the intersection between depression symptoms during the postpartum period, public health responses, pandemic-related interpersonal consequences, and the awareness of racial responses The qualitative participant-level results highlighted that among women receiving the intervention during the pandemic, the main facilitators were (1) the provision of creative problem-solving to attend the intervention, (2) BA helped deal with pandemic-related distress, (3) BA provided connection and support during the pandemic, and (4) telemedicine. The main barriers were (1) privacy, (2) greater pandemic-related stressors, and (3) limited activity owing to the pandemic	N/A

^a^T0: Time 0.

^b^T1: Time 1.

^c^T2: Time 2.

^d^T3: Time 3.

^e^BA: behavioral activation.

^f^N/A: not applicable.

^g^T4: Time 4.

## Discussion

### Principal Findings

This scoping review aimed to map the available literature on digital BA interventions administered during the perinatal period. The research questions guiding this work have focused on the interventions’ methodological underpinnings as well as on the interventions’ targets, referring to the specificities of the population considered as well as to the symptoms experienced by the latter.

Regarding research questions (4) *Were digital BA interventions able to influence psychosocial symptoms other than depression?* and (5) *Have they willingly been used to influence symptoms other than depression?* in line with expectations, they seem to be overall promising in reducing depression symptoms among postpartum women; however, owing to the limited literature available, it is not possible to draw definitive conclusions in this regard. Moreover, although an in-person BA intervention administered to pregnant women was effective in reducing anxiety symptoms [32], the sole concluded trial included in this review that had evaluated anxiety symptoms [42,46] reported no significant intervention effect. Accordingly, to date, it is not possible to draw complete conclusions on the generalizability of the efficacy of digital BA interventions on psychosocial variables (eg, anxiety and stress symptoms and quality of life) other than depression symptoms. This is mostly owing to the above-mentioned marginal investigation of these symptoms.

Referring, instead, to research question (6) *Are there differences in the BA interventions administered during the antenatal period versus the postnatal period?* it is noteworthy that of all the included studies, most [41-43,45,46] focused solely on the postnatal period by considering women who had recently given birth, whereas none specifically focused on pregnant women and, therefore, on the antenatal period. However, one of the included protocol studies [44] reported that it foresees the future inclusion of both antenatal and postnatal women to compare intervention efficacy as a function of the perinatal period during which it is deployed. Thus, overall, it is not possible to answer the research question focused on the efficacy of digital BA interventions deployed during the antenatal period versus the postnatal period. The lack of digital interventions administered during the antenatal period represents a limitation of the available literature in terms of both evidence-based treatments and prevention programs. This is relevant, especially considering the high prevalence of depressive symptoms during pregnancy (between 15% and 65% [50]) and of the interrelated [51,52] anxiety (between 18% and 24% [53]) and stress symptoms (low-moderate symptoms level 78% [54]). Altogether, these hinder women’s quality of life [55], with repercussions on the child’s development and well-being [56-58], while further significantly increasing the risk of postpartum depression [8,59]. These results might be particularly insightful if read through the lens of the Stepped Care model [60]. This specific approach to health care advocates for a highly collaborative approach among primary and secondary mental health services to choose and deliver the most appropriate treatment, ranging from primary low-burden care to more intensive and specialized care on the basis of patients’ specific level of need and distress, which should ultimately improve access to health care for patients on one hand and, on the other hand, the efficiency and cost-effectiveness of treatments themselves [60]. Within this theoretical framework, prevention programs can be regarded as primary health services; therefore, research on the feasibility and effectiveness of digital interventions within the perinatal period should be encouraged; such treatments are expected to be of great benefit for both women in the perinatal period and the health care system on the whole, as they should foster scalability and thereby reduce health care costs [61,62].

Similarly, with regard to overcoming the broader barriers already reported in the literature, limiting access to mental health care programs (eg, time constraints, lack of information about services, and social stigma [18-20]), the creation and implementation of acceptable and effective digital interventions would also be advantageous in terms of both time and expenses for both the patients and the health care specialists. Moreover, through the advent of social media platforms and the possibility to create ad hoc websites, digital interventions can be made known and spread with greater ease, thereby allowing the extension of care to patients living in hard-to-reach areas. These same premises might also be associated with a reduction in the social stigma surrounding health care seeking by, on the one hand, normalizing the reliance on psychological programs and, on the other hand, allowing the maintenance of privacy within one’s social environment. This review also attempted to identify the logistic, social, and pragmatic barriers specifically associated with digital BA interventions. Focusing, instead, on research question (3) *What were the main barriers, including both participants’ concerns and issues given by the digital tool itself, in implementing BA interventions through digital means?* the data reported here do not allow to gather insights or formulate suggestions regarding the existence and potential overcoming of barriers within the context of digital BA interventions during the perinatal period. In fact, only 1 study [47] specifically addressed the intervention’s barriers, raising some concerns about the maintenance of privacy during the intervention; however, this might be strictly linked to the use of telemedicine in general rather than on digital BA interventions specifically.

Regarding the remaining research questions, namely, (2) *Has a specific BA protocol been followed? if so, on what BA protocol were the interventions based? [39]* and (1) *How have BA interventions been structured (eg, intervention length, number of modules, topics considered, and guided versus unguided interventions) to be administered through digital means?* it is worth highlighting that all interventions were based on validated BA protocols [63], but no extensive explanation of the changes made to the original protocols were reported. Similarly, scarce information was reported on the role of the guidance in the guided interventions as well as on the specificities of the digital means used to administer the interventions. This limits the reproducibility of studies as well as the advancement of the matter, as it restricts the possibility of identifying the benefits and limitations of the different digital BA interventions. Likewise, all the included studies reported limited information on how the interventions’ content and home assignments were implemented and deployed. However, a thorough reporting of the choices made on the intervention content and structuring, of the specificities of the technological components used to implement the intervention, and of the potential drawbacks that might have emerged from the trials is needed to foster information exchange within the scientific community. This is particularly relevant, considering their value for the feasibility and efficacy of the intervention as well as for overall usability. Including such information in trial reports would ultimately contribute to the broader research field. In line with this thought, the work by O’Mahen et al [41,42] is noteworthy with regard to the investigation of the feasibility of interventions, as they reported refining their intervention structuring in line with participants’ feedback and then retesting its efficacy. In this regard, although none of the studies provided reasons for choosing a certain number of sessions composing the interventions, O’Mahen et al [41,42] reported reducing the number of core sessions while including some optional modules [42]—a decision based on participants’ feedback stating that they could not “keep up” with the intervention [41]. This particular mode of action is what seems to be lacking and should instead be promoted, as it provides information on the interventions’ specific structures, thereby favoring scientific exchange and, ultimately, the advancement of the matter. Future studies might benefit from including qualitative evaluations of the interventions’ experience and overall usability, for instance, by foreseeing the use of semistructured interviews at the end of the intervention [64]. In this regard, it should be stressed that usability was not evaluated by any of the concluded research studies included in this review, although the criticality of adherence to interventions was highlighted as it emerged in previous studies [65,66]. Usability is conceptualized as the output of the interaction between the user and the tools (eg, website) used [67] and includes 5 main concepts [68]: ease of use, intended as learnability, experienced by users learning how to use a digital tool; the efficiency with which users interact with the digital tool; the memorability of how to use a digital tool to which the user has been already exposed; the errors users make, intended as the number of trials needed to make a certain action correctly; and the perceived users’ satisfaction with the user experience. The latter further includes aspects relevant to user engagement, including the affective, cognitive, and behavioral response of the user to the digital tool [69]. In this regard, Bagnall [46], referring to the nonpredictive effect of intervention adherence on the intervention outcomes, discussed that beyond adherence, it might indeed be the level of engagement with the intervention, intended as behavioral participation, to be particularly relevant in influencing intervention efficacy. In line with this, O’Mahen et al [42] highlighted that intervention acceptability as well as adherence, as reported by women, was favored by the convenient delivery of the intervention’s content and the ease felt in following the program, which are all aspects relevant to usability. This further highlights the importance of accounting for usability facets when investigating the efficacy of digital interventions, which emerged in this review as a strong limitation of the literature on digital BA interventions deployed during the perinatal period. Future studies should, therefore, more carefully and consistently account for and thoroughly explain the results of the user experience while also accounting for user engagement [70].

Overall, the information retrieved through this scoping review does not allow to provide specific suggestions for the implementation of interventions. However, the gaps identified in the available literature can be useful for refining existing protocols and supporting the design of future interventions. Specifically, there has emerged a need to substantially investigate both the usability and user engagement associated with these digital interventions to better understand and refine them. The human-computer interaction aspects of these interventions, as of today, seem only marginally considered but should instead be central in future research, as they define the “setting” in which interventions take place. It should be noted that the existing literature does provide guidelines [70] or models [71] that should be followed in future trials to support adherence and engagement, as they are pivotal for the efficacy of interventions. Moreover, greater flexibility in the structuring of interventions (ie, the number of sessions and time required to complete each session) is recommended, as it would support acceptability. These aspects might be particularly valuable, considering the high attrition rate of these interventions, which had already been pointed out by existing literature and was coherently reported by the included studies as well [66].

### Conclusions

Taken together, the available literature examined was useful in highlighting the potential, and also the immaturity, of the research field on digital BA interventions, while promptly stressing the need to develop interventions specifically targeting pregnant women. To address this gap, future research should focus on addressing such limitations, paying particular attention to the investigation of the feasibility, acceptability, and usability of interventions. Moreover, future studies should invest in the development and evaluation of digital prevention programs deployed during pregnancy.

## Appendix

Multimedia Appendix 1 Supplementary materials.

## Abbreviations

ART: assisted reproductive technique
BA: behavioral activation
PRISMA: Preferred Reporting Items for Systematic Reviews and Meta-Analysis
PRISMA-ScR: Preferred Reporting Items for Systematic Reviews and Meta-Analysis extension for Scoping Reviews
RCT: randomized controlled trial
