# Supplementary material for: Digital Behavioral Activation Interventions During the Perinatal Period: Scoping Review
Source: JMIR Pediatr Parent. 2023 Feb 28;6:e40937. doi: 10.2196/40937 (PMC10015349; doi:10.2196/40937)
Supplement: Multimedia Appendix 1 [file pediatrics_v6i1e40937_app1.docx]

**Supplementa****ry Materials**

**Table S1**

**Reporting Checklist**

Preferred Reporting Items for Systematic reviews and Meta-Analyses extension for Scoping Reviews (PRISMA-ScR) Checklist [1]

| **SECTION** | **ITEM** | **PRISMA-ScR CHECKLIST ITEM** | **REPORTED ON PAGE #** |
| --- | --- | --- | --- |
| **TITLE** | | | |
| Title | 1 | Identify the report as a scoping review. | 1 |
| **ABSTRACT** | | | |
| Structured summary | 2 | Provide a structured summary that includes (as applicable): background, objectives, eligibility criteria, sources of evidence, charting methods, results, and conclusions that relate to the review questions and objectives. | 2 |
| **INTRODUCTION** | | | |
| Rationale | 3 | Describe the rationale for the review in the context of what is already known. Explain why the review questions/objectives lend themselves to a scoping review approach. | 3-5 |
| Objectives | 4 | Provide an explicit statement of the questions and objectives being addressed with reference to their key elements (e.g., population or participants, concepts, and context) or other relevant key elements used to conceptualize the review questions and/or objectives. | 5 |
| **METHODS** | | | |
| Protocol and registration | 5 | Indicate whether a review protocol exists; state if and where it can be accessed (e.g., a Web address); and if available, provide registration information, including the registration number. | n.a. |
| Eligibility criteria | 6 | Specify characteristics of the sources of evidence used as eligibility criteria (e.g., years considered, language, and publication status), and provide a rationale. | 6 |
| Information sources* | 7 | Describe all information sources in the search (e.g., databases with dates of coverage and contact with authors to identify additional sources), as well as the date the most recent search was executed. | 6 |
| Search | 8 | Present the full electronic search strategy for at least 1 database, including any limits used, such that it could be repeated. | 6-7 |
| Selection of sources of evidence† | 9 | State the process for selecting sources of evidence (i.e., screening and eligibility) included in the scoping review. | 6-7 |
| Data charting process‡ | 10 | Describe the methods of charting data from the included sources of evidence (e.g., calibrated forms or forms that have been tested by the team before their use, and whether data charting was done independently or in duplicate) and any processes for obtaining and confirming data from investigators. | 6-7 |
| Data items | 11 | List and define all variables for which data were sought and any assumptions and simplifications made. | 6-7 |
| Critical appraisal of individual sources of evidence§ | 12 | If done, provide a rationale for conducting a critical appraisal of included sources of evidence; describe the methods used and how this information was used in any data synthesis (if appropriate). | n.a. |
| Synthesis of results | 13 | Describe the methods of handling and summarizing the data that were charted. | n.a. |
| **RESULTS** | | | |
| Selection of sources of evidence | 14 | Give numbers of sources of evidence screened, assessed for eligibility, and included in the review, with reasons for exclusions at each stage, ideally using a flow diagram. | 7 & Figure 1 |
| Characteristics of sources of evidence | 15 | For each source of evidence, present characteristics for which data were charted and provide the citations. | 7-10 &  Table 1, Table 2, Table 3, table 4, Table S3 and Table S4 (supplementary materials), Figure 2. |
| Critical appraisal within sources of evidence | 16 | If done, present data on critical appraisal of included sources of evidence (see item 12). | n.a. |
| Results of individual sources of evidence | 17 | For each included source of evidence, present the relevant data that were charted that relate to the review questions and objectives. | 7-10 & Table 1, Table 2, Table 3, Table 4, Figure 2, Table S4 and Table S5 (supplementary materials) |
| Synthesis of results | 18 | Summarize and/or present the charting results as they relate to the review questions and objectives. | 10-12 |
| **DISCUSSION** | | | |
| Summary of evidence | 19 | Summarize the main results (including an overview of concepts, themes, and types of evidence available), link to the review questions and objectives, and consider the relevance to key groups. | 12-15 |
| Limitations | 20 | Discuss the limitations of the scoping review process. | 12-15 |
| Conclusions | 21 | Provide a general interpretation of the results with respect to the review questions and objectives, as well as potential implications and/or next steps. | 15 |
| **FUNDING** | | | |
| Funding | 22 | Describe sources of funding for the included sources of evidence, as well as sources of funding for the scoping review. Describe the role of the funders of the scoping review. | n.a. |

**Table S2**

Reports excluded after databases search, following the first title and abstract screening

| **N** | **Author, year** | **Title** |
| --- | --- | --- |
| 1 | [2] | Web-based surveillance of public information needs for informing preconception interventions |
| 2 | [3] | Effect of Gestational Diabetes Mellitus History on Future Pregnancy Behaviors: The Mutaba'ah Study |
| 3 | [4] | Parental practices, preferences, skills and attitudes on food consumption of pre-school children: Results from Nutriscience Project |
| 4 | [5] | Effectiveness of Internet-Based Electronic Technology Interventions on Breastfeeding Outcomes: Systematic Review |
| 5 | [6] | Adolescents' physical activity and sedentary behaviour in Indonesia during the COVID-19 pandemic: a qualitative study of mothers' perspectives |
| 6 | [7] | Health Communication in COVID-19 Era: Experiences from the Italian VaccinarSì Network Websites |
| 7 | [8] | Interest in web-based treatments for postpartum anxiety: an exploratory survey |
| 8 | [9] | Computer- or web-based interventions for perinatal mental health: A systematic review |
| 9 | [10] | The Men's Safer Sex project: intervention development and feasibility randomised controlled trial of an interactive digital intervention to increase condom use in men |
| 10 | [11] | Women's experiences of internet-delivered Cognitive Behaviour Therapy (iCBT) for Fear of Birth |
| 11 | [12] | Online platform for healthy weight loss in adults with overweight and obesity - the "POEmaS" project: a randomized controlled trial |
| 12 | [13] | Personalized Web-Based Weight Loss Behavior Change Program With and Without Dietitian Online Coaching for Adults With Overweight and Obesity: Randomized Controlled Trial |
| 13 | [14] | Web-Based Intervention for Women With Type 1 Diabetes in Pregnancy and Early Motherhood: Critical Analysis of Adherence to Technological Elements and Study Design |
| 14 | [15] | A Password-Protected Web Site for Mothers Expressing Milk for Their Preterm Infants |
| 15 | [16] | Internet-based interpersonal psychotherapy for stress, anxiety, and depression in prenatal women: study protocol for a pilot randomized controlled trial |
| 16 | [17] | Postnatal Exercise Partners Study (PEEPS): a pilot randomized trial of a dyadic physical activity intervention for postpartum mothers and a significant other |
| 17 | [18] | Online participatory intervention to promote and support exclusive breastfeeding: Randomized clinical trial |
| 18 | [19] | Identification of Risk Factors Associated with Obesity and Overweight-A Machine Learning Overview |
| 19 | [20] | Appraisal of systematic reviews on interventions for postpartum depression: systematic review |
| 20 | [21] | Medical Help-Seeking Strategies for Perinatal Women With Obstetric and Mental Health Problems and Changes in Medical Decision Making Based on Online Health Information: Path Analysis |
| 21 | [22] | An internet-based mind/body intervention to mitigate distress in women experiencing infertility: A randomized pilot trial |
| 22 | [23] | Targeting the Infant Gut Microbiota Through a Perinatal Educational Dietary Intervention: Protocol for a Randomized Controlled Trial |
| 23 | [24] | 'Reducing Delays In Vaccination' (REDIVAC) trial: a protocol for a randomised controlled trial of a web-based, individually tailored, educational intervention to improve timeliness of infant vaccination |
| 24 | [25] | Protocol for a randomised trial evaluating a preconception-early childhood telephone-based intervention with tailored e-health resources for women and their partners to optimise growth and development among children in Canada: a Healthy Life Trajectory Initiative (HeLTI Canada) |
| 25 | [26] | Rationale and design of ePPOP-ID: a multicenter randomized controlled trial using an electronic-personalized program for obesity in pregnancy to improve delivery |
| 26 | [27] | "You Are on the Right Track With the App:" Qualitative Analysis of Mobile Phone Use and User Feedback Regarding Mobile Phone Sexual Risk Assessments for HIV Prevention Research |
| 27 | [28] | High cellphone use associated with greater risk of depression among young women aged 15-24 years in Soweto and Durban, South Africa |
| 28 | [29] | Investigating Sociodemographic Factors and HIV Risk Behaviors Associated With Social Networking Among Adolescents in Soweto, South Africa: A Cross-Sectional Survey |
| 29 | [30] | Selective dietary supplementation in early postpartum is associated with high resilience against depressed mood |
| 30 | [31] | Informing Active Play and Screen Time Behaviour Change Interventions for Low Socioeconomic Position Mothers of Young Children: What Do Mothers Want? |
| 31 | [32] | Experiences of using a digital type 2 diabetes prevention application designed to support women with previous gestational diabetes |
| 32 | [33] | Perceptions and intervention preferences of Moroccan adolescents, parents, and teachers regarding risks and protective factors for risky sexual behaviors leading to sexually transmitted infections in adolescents: qualitative findings |
| 33 | [34] | Be a Mom: Formative Evaluation of a Web-Based Psychological Intervention to Prevent Postpartum Depression |
| 34 | [35] | Internet delivered cognitive behavior therapy for antenatal depression: A randomised controlled trial |
| 35 | [36] | Global, regional, and national progress towards Sustainable Development Goal 3.2 for neonatal and child health: all-cause and cause-specific mortality findings from the Global Burden of Disease Study 2019 |
| 36 | [37] | Acceptability of financial incentives for breastfeeding: thematic analysis of readers' comments to UK online news reports |
| 37 | [38] | Interventions to improve or facilitate linkage to or retention in pre-ART (HIV) care and initiation of ART in low- and middle-income settings--a systematic review |
| 38 | [39] | The theory, development, and implementation of an e-intervention to prevent excessive gestational weight gain: e-Moms Roc |
| 39 | [40] | Does Usage of an eHealth Intervention Reduce the Risk of Excessive Gestational Weight Gain? Secondary Analysis From a Randomized Controlled Trial |
| 40 | [41] | Developmental effects of early immune stress on aggressive, socially reactive, and inhibited behaviors |
| 41 | [42] | A Social Media Peer Group Intervention for Mothers to Prevent Obesity and Promote Healthy Growth from Infancy: Development and Pilot Trial |
| 42 | [43] | A whole family-based physical activity promotion intervention: findings from the families reporting every step to health (FRESH) pilot randomised controlled trial |
| 43 | [44] | Whole family-based physical activity promotion intervention: the Families Reporting Every Step to Health pilot randomised controlled trial protocol |
| 44 | [45] | Internet-based support for infertile patients: a randomized controlled study |
| 45 | [46] | High maternal self-efficacy is associated with meeting Institute of Medicine gestational weight gain recommendations |
| 46 | [47] | A Mobile Application for Monitoring and Management of Depressed Mood in a Vulnerable Pregnant Population |
| 47 | [48] | Reduction in physical activity significantly increases depression and anxiety in the perinatal period: a longitudinal study based on a self-report digital assessment tool |
| 48 | [49] | Sertraline concentrations in pregnant women are steady and the drug transfer to their infants is low |
| 49 | [50] | MAGDALENA: study protocol of a randomised, placebo-controlled trial on cognitive development at 2 years of age in children exposed to SSRI in utero |
| 50 | [51] | Decreased levels of total immunoglobulin in children with autism are not a result of B cell dysfunction |
| 51 | [52] | Ethnic differences in risk factors for obesity in New Zealand infants |
| 52 | [53] | Feasibility of conducting an early pregnancy diet and lifestyle e-health intervention: the Pregnancy Lifestyle Activity Nutrition (PLAN) project |
| 53 | [54] | Mobile Phone-Based Behavioral Interventions in Pregnancy to Promote Maternal and Fetal Health in High-Income Countries: Systematic Review |
| 54 | [55] | Mobile Health for Perinatal Depression and Anxiety: Scoping Review |
| 55 | [56] | Be Healthe for Your Heart: A Pilot Randomized Controlled Trial Evaluating a Web-Based Behavioral Intervention to Improve the Cardiovascular Health of Women with a History of Preeclampsia |
| 56 | [57] | Mindfulness-based cognitive therapy for chronic depression |
| 57 | [58] | Participation of African Americans in e-Health and m-Health Studies: A Systematic Review |
| 58 | [59] | A three-component cognitive behavioural lifestyle program for preconceptional weight-loss in women with polycystic ovary syndrome (PCOS): a protocol for a randomized controlled trial |
| 59 | [60] | Effectiveness of music therapy: a summary of systematic reviews based on randomized controlled trials of music interventions |
| 60 | [61] | A qualitative investigation of optimal perinatal health: the perspectives of south Asian grandmothers living in southern Ontario, Canada |
| 61 | [62] | A proof‐of‐concept pilot randomized comparative trial of brief internet‐based compassionate mind training and cognitive‐behavioral therapy for perinatal and intending to become pregnant women |
| 62 | [63] | Comparing Brief Internet-Based Compassionate Mind Training and Cognitive Behavioral Therapy for Perinatal Women: Study Protocol for a Randomized Controlled Trial |
| 63 | [64] | Brief Internet-Based Intervention Reduces Posttraumatic Stress and Prolonged Grief in Parents after the Loss of a Child during Pregnancy: A Randomized Controlled Trial |
| 64 | [65] | A web-based pedometer programme in women with a recent history of gestational diabetes |
| 65 | [66] | Determinants of Pregnant Women's Online Self-Regulatory Activities for Appropriate Gestational Weight Gain |
| 66 | [67] | Two-Year Outcomes of the Enabling Mothers to Prevent Pediatric Obesity Through Web-Based Education and Reciprocal Determinism (EMPOWER) Randomized Control Trial |
| 67 | [68] | A Feasibility and Efficacy Randomized Controlled Trial of an Online Preventative Program for Childhood Obesity: Protocol for the EMPOWER Intervention |
| 68 | [69] | Mindfulness for pregnancy: A randomised controlled study of online mindfulness during pregnancy |
| 69 | [70] | Global Prevalence of Fetal Alcohol Spectrum Disorder Among Children and Youth: A Systematic Review and Meta-analysis |
| 70 | [71] | Effectiveness of digital psychotherapeutic intervention among perinatal women: A systematic review and meta-analysis of randomized controlled trials |
| 71 | [72] | Key Lessons and Impact of the Growing Healthy mHealth Program on Milk Feeding, Timing of Introduction of Solids, and Infant Growth: Quasi-Experimental Study |
| 72 | [73] | Web-based interventions for prevention and treatment of perinatal mood disorders: a systematic review |
| 73 | [74] | A Smartphone App to Restore Optimal Weight (SPAROW) in Women With Recent Gestational Diabetes Mellitus: Randomized Controlled Trial |
| 74 | [75] | Sources of information about gestational weight gain, diet and exercise among Brazilian immigrant women living in the USA: a cross-sectional study |
| 75 | [76] | Exploring how Brazilian immigrant mothers living in the USA obtain information about physical activity and screen time for their preschool-aged children: a qualitative study |
| 76 | [77] | Mindfulness-based stress reduction for family carers of people with dementia |
| 77 | [78] | Using Community Engagement to Develop a Web-Based Intervention for Latinos about the HPV Vaccine |
| 78 | [79] | Passive sensing on mobile devices to improve mental health services with adolescent and young mothers in low-resource settings: the role of families in feasibility and acceptability |
| 79 | [80] | Engaging family members in maternal, infant and young child nutrition activities in low- and middle-income countries: A systematic scoping review |
| 80 | [81] | Risk factors in autism: Thinking outside the brain |
| 81 | [82] | Preconceptional health behavior change in women with overweight and obesity: prototype for SMART strong healthy women intervention |
| 82 | [83] | Continuous Subcutaneous Insulin Infusion (CSII) Pumps for Type 1 and Type 2 Adult Diabetic Populations: An Evidence-Based Analysis |
| 83 | [84] | Effect of Lifestyle Coaching Including Telemonitoring and Telecoaching on Gestational Weight Gain and Postnatal Weight Loss: A Systematic Review |
| 84 | [85] | Pokémon GO Within the Context of Family Health: Retrospective Study |
| 85 | [86] | Targeted mass media interventions promoting healthy behaviours to reduce risk of non-communicable diseases in adult, ethnic minorities |
| 86 | [87] | The effectiveness of telemedicine interventions to address maternal depression: A systematic review and meta-analysis |
| 87 | [88] | A Qualitative Evaluation of Telephone-Based Cognitive-Behavioral Therapy for Postpartum Mothers |
| 88 | [89] | The effect of a telephone-based cognitive behavioral therapy on quality of life: a randomized controlled trial |
| 89 | [90] | Interventions to Prevent Perinatal Depression: A Systematic Evidence Review for the U.S. Preventive Services Task Force |
| 90 | [91] | Point prevalence survey of antibiotic use and resistance at a referral hospital in Kenya: findings and implications |
| 91 | [92] | The effectiveness of an online intervention in preventing excessive gestational weight gain: the e-moms roc randomized controlled trial |
| 92 | [93] | Active Mothers Postpartum: a randomized controlled weight-loss intervention trial |
| 93 | [94] | Effectiveness of a digital dietary intervention program targeting young adults before parenthood: protocol for the PREPARED randomised controlled trial |
| 94 | [95] | Predictors of changes in adolescents' consumption of fruits, vegetables and energy-dense snacks |
| 95 | [96] | 'Fit Moms/Mamás Activas' internet-based weight control program with group support to reduce postpartum weight retention in low-income women: study protocol for a randomized controlled trial |
| 96 | [97] | Effect of an Internet-Based Program on Weight Loss for Low-Income Postpartum Women: A Randomized Clinical Trial |
| 97 | [98] | Effect of a behavioural intervention in obese pregnant women (the UPBEAT study): a multicentre, randomised controlled trial |
| 98 | [99] | Wearable Digital Sensors to Identify Risks of Postpartum Depression and Personalize Psychological Treatment for Adolescent Mothers: Protocol for a Mixed Methods Exploratory Study in Rural Nepal |
| 99 | [100] | Engagement and Weight Loss in a Web and Mobile Program for Low-Income Postpartum Women: Fit Moms/Mamás Activas |
| 100 | [101] | Opioid effect on the autonomic nervous system in a fetal sheep model |
| 101 | [102] | Experiences, Attitudes, and Needs of Users of a Pregnancy and Parenting App (Baby Buddy) During the COVID-19 Pandemic: Mixed Methods Study |
| 102 | [103] | Investigating partner involvement in pregnancy and identifying barriers and facilitators to participating as a couple in a digital healthy eating and physical activity intervention |
| 103 | [104] | Depression and health behaviors in women with Peripartum Cardiomyopathy |
| 104 | [105] | Impact of the Growing Healthy mHealth Program on Maternal Feeding Practices, Infant Food Preferences, and Satiety Responsiveness: Quasi-Experimental Study |
| 105 | [106] | Effects of Exercise-Based Interventions on Neonatal Outcomes: A Meta-Analysis of Randomized Controlled Trials |
| 106 | [107] | Effectiveness of a Smartphone App to Promote Healthy Weight Gain, Diet, and Physical Activity During Pregnancy (HealthyMoms): Randomized Controlled Trial |
| 107 | [108] | Internet-based stress management for women with preterm labour--a case-based experience report |
| 108 | [109] | Influence of family environment on long-term psychosocial functioning of adolescents with juvenile fibromyalgia |
| 109 | [110] | Implementation and Effectiveness of Nonspecialist-Delivered Interventions for Perinatal Mental Health in High-Income Countries A Systematic Review and Meta-analysis |
| 110 | [111] | A complex behavioural change intervention to reduce the risk of diabetes and prediabetes in the pre-conception period in Malaysia: study protocol for a randomised controlled trial |
| 111 | [112] | Differences in Utilization of Perinatal Psychiatric Teleconsultation Line Between Primary Care and Mental Health Providers |
| 112 | [113] | A review of web-based technology in behavioural activation |
| 113 | [114] | Pre-Conception Interventions for Subfertile Couples Undergoing Assisted Reproductive Technology Treatment: Modeling Analysis |
| 114 | [115] | Effect of behavioural-educational intervention on sleep for primiparous women and their infants in early postpartum: multisite randomised controlled trial |
| 115 | [116] | Be Healthe for Your Heart: Protocol for a Pilot Randomized Controlled Trial Evaluating a Web-Based Behavioral Intervention to Improve the Cardiovascular Health of Women With a History of Preeclampsia |
| 116 | [117] | Physical activity during pregnancy and the role of theory in promoting positive behavior change: A systematic review |
| 117 | [118] | Maternal stress and diet may influence affective behavior and stress-response in offspring via epigenetic regulation of central peptidergic function |
| 118 | [119] | Guided Internet-Based Parent Training for Challenging Behavior in Children With Fetal Alcohol Spectrum Disorder (Strongest Families FASD): Study Protocol for a Randomized Controlled Trial |
| 119 | [120] | Gender-related influences on adherence to advice and treatment-seeking guidance for infants and young children post-hospital discharge in Bangladesh |
| 120 | [121] | Informing abortion counseling: an examination of evidence-based practices used in emotional care for other stigmatized and sensitive health issues |
| 121 | [122] | Efficacy of an internet-based cognitive behavioral stress management training in women with idiopathic preterm labor: A randomized controlled intervention study |
| 122 | [123] | Home-based telemonitoring versus hospital admission in high risk pregnancies: a qualitative study on women's experiences |
| 123 | [124] | eHealth as the Next-Generation Perinatal Care: An Overview of the Literature |
| 124 | [125] | Cross-sectional associations between maternal parenting styles, physical activity and screen sedentary time in children |
| 125 | [126] | Personalized Web-Based Advice in Combination With Well-Child Visits to Prevent Overweight in Young Children: Cluster Randomized Controlled Trial |
| 126 | [127] | Effects of Three Different Family-Based Interventions in Overweight and Obese Children: The "4 Your Family" Randomized Controlled Trial |
| 127 | [128] | A review of community-based participatory research in child health |
| 128 | [129] | Telemedicine in perinatal mental health: perspectives |
| 129 | [130] | Postpartum Mental Health Care for Mothers of Multiples: A Qualitative Study of New Mothers' Treatment Preferences |
| 130 | [131] | Online Interventions for Postpartum Depression |
| 131 | [132] | Variations in health behaviors among pregnant women during the COVID-19 pandemic |
| 132 | [133] | Pregnant women's perceptions of weight gain, physical activity, and nutrition using Theory of Planned Behavior constructs |
| 133 | [134] | Gamifying Breastfeeding for Fathers: Process Evaluation of the Milk Man Mobile App |
| 134 | [135] | Safe sext: adolescents' use of technology to communicate about sexual health with dating partners |
| 135 | [136] | A prospective cohort study of physical activity and time to pregnancy |
| 136 | [137] | The Influence of Web-Based Tools on Maternal and Neonatal Outcomes in Pregnant Adolescents or Adolescent Mothers: Mixed Methods Systematic Review |
| 137 | [138] | Healthcare interventions for the prevention and control of gestational diabetes mellitus in China: a scoping review |
| 138 | [139] | Fathers' involvement during pregnancy and childbirth: An integrative literature review |
| 139 | [140] | Effectiveness of Telehealth Interventions for Women With Postpartum Depression: Systematic Review and Meta-analysis |
| 140 | [141] | Proceedings of the 3rd IPLeiria's International Health Congress : Leiria, Portugal. 6-7 May 2016 |
| 141 | [142] | Proceedings of the 8th Annual Conference on the Science of Dissemination and Implementation : Washington, DC, USA. 14-15 December 2015 |

**Table S3**

Reports excluded after databases search, following the full-text screening, with reasons for exclusion

| **Author, year** | **Title** | **Reason for exclusion** |
| --- | --- | --- |
| [143] | Feasibility and Acceptability of a Web-Based Treatment with Telephone Support for Postpartum Women with Anxiety: Randomized Controlled Trial | The study did not evaluate a BA intervention |
| [144] | Women's experiences of internet-delivered Cognitive Behaviour Therapy (iCBT) for Fear of Birth | The study did not evaluate a BA intervention |
| [145] | The INTER-ACT E-Health Supported Lifestyle Intervention Improves Postpartum Food Intake and Eating Behavior, but Not Physical Activity and Sedentary Behavior-A Randomized Controlled Trial | The study did not evaluate a BA intervention |
| [146] | INTER-ACT: prevention of pregnancy complications through an e-health driven interpregnancy lifestyle intervention - study protocol of a multicentre randomised controlled trial | The study did not evaluate a BA intervention |
| [147] | Web-Based Intervention for Postpartum Depression: Formative Research and Design of the MomMoodBooster Program | The study did not evaluate a BA intervention |
| [148] | MomMoodBooster web-based intervention for postpartum depression: Feasibility trial results | The study did not evaluate a BA intervention |
| [149] | An Internet-Based Intervention (Mamma Mia) for Postpartum Depression: Mapping the Development from Theory to Practice | The study did not evaluate a BA intervention |
| [150] | Internet cognitive behavioral therapy for women with postnatal depression: A randomized controlled trial of MumMoodBooster | The study did not evaluate a BA intervention |
| [151] | A Qualitative Evaluation of Telephone-Based Cognitive-Behavioral Therapy for Postpartum Mothers | The study did not evaluate a BA intervention |
| [152] | Randomized Controlled Trial of an Internet-Based Educational Intervention for Mothers With Mental Illnesses: An 18-Month Follow-Up | The study did not evaluate a BA intervention |
| [153] | Move my mood: Development and evaluation of a mobile mental health self-help app using behavioral activation for women with postpartum depression | No full text available |
| [154] | The effect of internet‐based guided self‐help cognitive‐behavioral therapies on Iranian women's psychological symptoms and preferred method of childbirth | The study did not evaluate a BA intervention |
| [155] | Treating postpartum depression in rural veterans using internet delivered CBT: Program evaluation of MomMoodBooster | The study did not evaluate a BA intervention |
| [156] | Telecare for women with postpartum depression | No full text available |

**Table S4**

Reports excluded after screening of key journals and reference lists

| **Author, year** | **Title** | **Reason for exclusion** |
| --- | --- | --- |
| [157] | C4-5: Behavioral Activation Therapy for Perinatal Depression: Preliminary Results from a Multi-site Randomized Trial | The BA intervention was not delivered via digital means |
| [158] | A Pragmatic Randomized Clinical Trial of Behavioral Activation for Depressed Pregnant Women | The BA intervention was not delivered via digital means |
| [159] | Internet delivered cognitive behavior therapy for antenatal depression: A randomised controlled trial | The study did not evaluate a BA intervention |
| [160] | Effect of the healthy MOMs lifestyle intervention on reducing depressive symptoms among pregnant Latinas | The BA intervention was not delivered via digital means |

**Table S5**

Studies’ main outcomes

| **Author, year** | **Maternal variables** | **Intervention evaluation** | **Child-related variables** |
| --- | --- | --- | --- |
| [161] | - Depression symptoms - Anxiety symptoms - Life functioning - Social support - Behavioral Activation | - Intervention adherence | n.a. |
| [162] | - Depression symptoms | - Feasibility - Acceptability | n.a. |
| [163] | - Depression symptoms - Anxiety symptoms - Life functioning - Social support - Mother-child bonding | n.a. | n.a. |
| [164] | - Depression symptoms - Social support - Behavioral Activation - Life functioning | - Intervention adherence | n.a. |
| [165] | - Depression symptoms - Parental stress - Mother-child bonding - Quality of Life - Maternal healthcare use | - Feasibility - Acceptability | - Psychological aggression - Physical assault |
| [166] | - Depression symptoms - Anxiety symptoms - Social support - Quality of Life - Behavioral Activation - Maternal healthcare use | - Intervention adherence - Therapy quality - Usability | - Psychological stimulation - Mental development |
| [167] | - Depression symptoms | n.a. | n.a. |

*Note*. n.a. = not applicable

**Table S6**

Sample's characteristics

| **Author, year** |  | **Age**  **M (SD)** | **Ethnicity**  **N (%)** | **Occupation**  **N (%)** | **Marital status**  **N (%)** | **Educational level**  **N (%)** | **Number of children**  **N (%)** | **Income**  **N (%)** |
| --- | --- | --- | --- | --- | --- | --- | --- | --- |
| [162] | **EG** | 32,3 (4,7) | n.r. | - Homemaker/maternity or disability leave: 122 (73,9) - Full or part-time employment: 41 (24,9)   Student or volunteer: 2 (1,2) | - Married/cohabiting: 123 (91,8) - Divorced/separated: 5 (3,7)   Not in a relationship now: 6 (4,5) | - None; 3 (1,8) - Secondary: 38 (23,3) - Post-16: 42 (25,8)   First degree of Higher degree: 80 (49,1) | - 1: 69 (41,8) - 2: 71 (43) - 3: 15 (9,1)   ≥4: 10 (6,1) | n.r. |
|  | **CG** | 32,2 (5,7) | n.r. | - Homemaker/maternity or disability leave: 75 (56,4 - Full or part-time employment: 57 (42,9)   Student or volunteer: 1 (0,8) | - Married/cohabiting: 157 (95,2) - Divorced/separated: 3 (1,8)   Not in a relationship now: 5 (3) | - None: 1 (0,8) - Secondary: 38 (29,0) - Post-16: 40 (30,5)   First degree of Higher degree: 52 (39,7) | - 1: 53 (39,6) - 2: 58 (43,3) - 3: 20 (14,9)   ≥4: 3 (2,2) | n.r |
| [161,163] | **EG** | n.r. | - White/British: 38 (92,6) - Asian: 1 (2,4) - Mixed white /African/ Caribbean: 0 - African: 0 - Other: 2 (4,8) | - Homemaker/maternity or disability leave: 32 (80,5) - Full or part-time employment: 3 (7,3) - Student or volunteer: 3 (7,3) | - Married/cohabiting: 38 (92,6) - Not in a relationship now: 3 (7,3) | - None: 1 (2,4) - Secondary: 4 (9,7) - Post-16 years: 13 (31,7) - Undergraduate degree: 13 (31,7) - Graduate degree: 10 (24,4) | - 1: 19 (46,3) - 2: 18 (43,9) - 3: 1 (2,4) - ≥4: 3 (7,6) | - <£10000 4 (9,2) - £10000–£19000: 6 (14,6) - £20000–£29999: 6 (14,6) - £30000–£39999: 5 (12,2) - £40000–£49999: 4 (9,8) - £50000–£59999: 6 (14,6) - £60000–£69999: 7 (17,1) - £70000–£79999: 2 (4,9) - £80000: n.r. |
|  | **CG** | n.r. | - White/British: 39 (92,9) - Asian: 0 - Mixed white /African/ Caribbean: 2 (4,8) - African: 1 (2,4) - Other: 0 | - Homemaker/maternity or disability leave: 32 (80,5) - Full or part-time employment: 6 (14,3) - Student or volunteer: 4 (9,6) | - Married/cohabiting: 38 (90,5) - Not in a relationship now: 4 (9,5) | - None: n.r. - Secondary: 11 (26,2) - Post-16 years: 10 (23,8) - Undergraduate degree: 12 (28,6) - Graduate degree 9: (21,4) | - 1: 16 (38,1) - 2: 16 (38,1) - 3: 9 (21,4) - ≥4: 1 (2,4) | - <£10000: 2 (4,8) - £10000–£19000: 10 (23,8) - £20000–£29999: 4 (9,5) - £30000–£39999: 10 (23,8) - £40000–£49999: 7 (16,7) - £50000–£59999: 2 (4,8) - £60000–£69999: 3 (7,1) - £70000–£79999: 3 (7,1) - £80000: 1 (2,4) |
| [164] |  | n.r. | - White/British: 30 (92,6) - Other: n.r. | - Homemaker/maternity leave: 26 (80,5) - Student/full-time employment: 5 (14,6) | - Married/cohabiting: 28 (90,2) | - < Undergraduate degree: 13 (41,4) - Undergraduate/postgraduate degree: 18 (56,1) | - Median: 2 (range: 1-7) | - <£30,000: 8 (38,4) - £30-60,000: 12 (36,4) - >£60,000: 9 (25,2) |
| [167] |  | 32  (n.r.) | - White: 12 (52,2) - Black/African American: 2 (8,7) - Hispanic: 2 (8,7) - Multi race: 2 (8,7) - Asian: 2 (8,7) - No answer: 2 (8,7) - Other: 1 (4,3) | - Maternity leave: 8 (34,8) - Full-time employment: 6 (26,1) - Part-time employment: 3 (13) - Unemployed: 3 (13) - Student: 2 (8,7) - Homemaker: 1 (4,3) | - Married: 19 (82,6) - Single: 4 (23,5) - Dating: 1 (4,3) - No answer: 2 (8,7) | n.a. | n.a. | n.a. |

*Note*. EG = experimental group; CG = Control Group; n.r = not reported; n.a. = not applicable

**Table S7**

Interventions’ content specificities

| **Author, year** | **Original protocol** | **Amends to original protocol** | **Intervention structure** | **Intervention key content** |
| --- | --- | --- | --- | --- |
| [165] | Self-help booklet for postnatal depression (O’Mahen & Hopley, s.d.) | - Adapted for Japanese postpartum women | - One 30 minutes weekly session - Online assignments | - Psychoeducation - Identify avoidance patterns - Focus on meaningful activities - Foster support and communication - Relapse prevention |
| [162] | BA functional analytical framework (Addis & Martell, 2004) | - Adapted for postpartum women - Adapted to be delivered online - Included iterative input from stakeholders | - One 40 minutes weekly session - Online assignments - Sessions could be customized by selecting topics | - Psychoeducation - Identify avoidance patterns - Focus on meaningful activities - Contingency planning and problem-solving - Changes in roles - Support friendship with other mothers - Foster communication strategies - Ruminative thoughts - Relapse prevention |
| [161,163,164] | BA functional analytical framework (Addis & Martell, 2004) | - Same as O’Mahen et al., 2013 - Further modified intervention content and simplified its presentation based on the feedback from O’Mahen et al., 2013 study | - One weekly session plus one telephone session - 5 core sessions and a relapse prevention session plus 2 optional sessions to be chose from a list of 6 - Online interactive exercises | Core sessions:   - Psychoeducation - Identify avoidance patterns - Focus on meaningful activities - Contingency planning and problem-solving   Optional sessions:   - Motherhood - Foster support and communication - Changes in roles and relationships - Sleep - Ruminative thoughts, anxiety and fears |
| [166] | Alma Program for perinatal populations in Colorado and the Healthy Activity Program (Chowdhary et al., 2016; Patel et al., 2017) | n/r | - One 20-30 minutes weekly session - Offline assignments | - Psychoeducation - Identification of key values and focus on value-based activities - Activities monitoring and scheduling - Focus on interpersonal mechanisms - Support problem solving |
| [167] | Healthy Activity Program (Patel et al., 2017) | - Adapted to the perinatal population | - One 20-30 minutes weekly session - Offline assignments | - Identification of key values through a values card sort - Focus on interpersonal mechanisms and communication skills - Include relaxation techniques - Adapted to address pandemic related concerns |

*Note*. n/r = not reported

***Bibliography***

1. Tricco, A.C.; Lillie, E.; Zarin, W.; O’Brien, K.K.; Colquhoun, H.; Levac, D.; Moher, D.; Peters, M.D.J.; Horsley, T.; Weeks, L.; et al. PRISMA Extension for Scoping Reviews (PRISMA-ScR): Checklist and Explanation. *Annals of Internal Medicine* **2018**, *169*, 467–473, doi:10.7326/M18-0850.

2. D’Ambrosio, A.; Agricola, E.; Russo, L.; Gesualdo, F.; Pandolfi, E.; Bortolus, R.; Castellani, C.; Lalatta, F.; Mastroiacovo, P.; Tozzi, A.E. Web-Based Surveillance of Public Information Needs for Informing Preconception Interventions. *PLoS One* **2015**, *10*, e0122551, doi:10.1371/journal.pone.0122551.

3. Ali, N.; Aldhaheri, A.S.; Alneyadi, H.H.; Alazeezi, M.H.; Al Dhaheri, S.S.; Loney, T.; Ahmed, L.A. Effect of Gestational Diabetes Mellitus History on Future Pregnancy Behaviors: The Mutaba’ah Study. *Int J Environ Res Public Health* **2020**, *18*, E58, doi:10.3390/ijerph18010058.

4. Almeida, C.; Azevedo, J.; Gregório, M.J.; Barros, R.; Severo, M.; Padrão, P. Parental Practices, Preferences, Skills and Attitudes on Food Consumption of Pre-School Children: Results from Nutriscience Project. *PLoS One* **2021**, *16*, e0251620, doi:10.1371/journal.pone.0251620.

5. Almohanna, A.A.; Win, K.T.; Meedya, S. Effectiveness of Internet-Based Electronic Technology Interventions on Breastfeeding Outcomes: Systematic Review. *J Med Internet Res* **2020**, *22*, e17361, doi:10.2196/17361.

6. Andriyani, F.D.; Biddle, S.J.H.; De Cocker, K. Adolescents’ Physical Activity and Sedentary Behaviour in Indonesia during the COVID-19 Pandemic: A Qualitative Study of Mothers’ Perspectives. *BMC Public Health* **2021**, *21*, 1864, doi:10.1186/s12889-021-11931-1.

7. Arghittu, A.; Dettori, M.; Dempsey, E.; Deiana, G.; Angelini, C.; Bechini, A.; Bertoni, C.; Boccalini, S.; Bonanni, P.; Cinquetti, S.; et al. Health Communication in COVID-19 Era: Experiences from the Italian VaccinarSì Network Websites. *Int J Environ Res Public Health* **2021**, *18*, 5642, doi:10.3390/ijerph18115642.

8. Ashford, M.T.; Ayers, S.; Olander, E.K. Interest in Web-Based Treatments for Postpartum Anxiety: An Exploratory Survey. *J. Reprod. Infant Psychol.* **2017**, *35*, 394–409, doi:10.1080/02646838.2017.1320364.

9. Ashford, M.T.; Olander, E.K.; Ayers, S. Computer- or Web-Based Interventions for Perinatal Mental Health: A Systematic Review. *J. Affect. Disord.* **2016**, *197*, 134–146, doi:10.1016/j.jad.2016.02.057.

10. Bailey, J.V.; Webster, R.; Hunter, R.; Griffin, M.; Freemantle, N.; Rait, G.; Estcourt, C.; Michie, S.; Anderson, J.; Stephenson, J.; et al. The Men’s Safer Sex Project: Intervention Development and Feasibility Randomised Controlled Trial of an Interactive Digital Intervention to Increase Condom Use in Men. *Health Technol Assess* **2016**, *20*, 1–124, doi:10.3310/hta20910.

11. Baylis, R.; Ekdahl, J.; Haines, H.; Rubertsson, C. Women’s Experiences of Internet-Delivered Cognitive Behaviour Therapy (ICBT) for Fear of Birth. *WOMEN BIRTH* **2020**, *33*, e227–e233, doi:10.1016/j.wombi.2019.05.006.

12. Beleigoli, A.M.; Queiroz de Andrade, A.; Haueisen Diniz, M. de F.; Alvares, R.S.; Ribeiro, A.L. Online Platform for Healthy Weight Loss in Adults with Overweight and Obesity - the “POEmaS” Project: A Randomized Controlled Trial. *BMC Public Health* **2018**, *18*, 945, doi:10.1186/s12889-018-5882-y.

13. Beleigoli, A.; Andrade, A.Q.; Diniz, M.D.F.; Ribeiro, A.L. Personalized Web-Based Weight Loss Behavior Change Program With and Without Dietitian Online Coaching for Adults With Overweight and Obesity: Randomized Controlled Trial. *J Med Internet Res* **2020**, *22*, e17494, doi:10.2196/17494.

14. Berg, M.; Linden, K.; Adolfsson, A.; Sparud Lundin, C.; Ranerup, A. Web-Based Intervention for Women With Type 1 Diabetes in Pregnancy and Early Motherhood: Critical Analysis of Adherence to Technological Elements and Study Design. *J Med Internet Res* **2018**, *20*, e160, doi:10.2196/jmir.9665.

15. Blatz, M.; Dowling, D.; Underwood, P.W.; Bieda, A.; Graham, G. A Password-Protected Web Site for Mothers Expressing Milk for Their Preterm Infants. *Adv Neonatal Care* **2017**, *17*, 222–229, doi:10.1097/ANC.0000000000000365.

16. Bright, K.S.; Mughal, M.K.; Wajid, A.; Lane-Smith, M.; Murray, L.; Roy, N.; Van Zanten, S.V.; Mcneil, D.A.; Stuart, S.; Kingston, D. Internet-Based Interpersonal Psychotherapy for Stress, Anxiety, and Depression in Prenatal Women: Study Protocol for a Pilot Randomized Controlled Trial. *Trials* **2019**, *20*, 814, doi:10.1186/s13063-019-3897-z.

17. Carr, R.M.; Quested, E.; Stenling, A.; Thøgersen-Ntoumani, C.; Prestwich, A.; Gucciardi, D.F.; McVeigh, J.; Ntoumanis, N. Postnatal Exercise Partners Study (PEEPS): A Pilot Randomized Trial of a Dyadic Physical Activity Intervention for Postpartum Mothers and a Significant Other. *Health Psychol Behav Med* **2021**, *9*, 251–284, doi:10.1080/21642850.2021.1902815.

18. Cavalcanti, D.S.; Cabral, C.S.; de Toledo Vianna, R.P.; Osório, M.M. Online Participatory Intervention to Promote and Support Exclusive Breastfeeding: Randomized Clinical Trial. *Matern Child Nutr* **2019**, *15*, e12806, doi:10.1111/mcn.12806.

19. Chatterjee, A.; Gerdes, M.W.; Martinez, S.G. Identification of Risk Factors Associated with Obesity and Overweight-A Machine Learning Overview. *Sensors (Basel)* **2020**, *20*, E2734, doi:10.3390/s20092734.

20. Chow, R.; Huang, E.; Li, A.; Li, S.; Fu, S.Y.; Son, J.S.; Foster, W.G. Appraisal of Systematic Reviews on Interventions for Postpartum Depression: Systematic Review. *BMC Pregnancy Childbirth* **2021**, *21*, 18, doi:10.1186/s12884-020-03496-5.

21. Chung, K.; Cho, H.Y.; Kim, Y.R.; Jhung, K.; Koo, H.S.; Park, J.Y. Medical Help-Seeking Strategies for Perinatal Women With Obstetric and Mental Health Problems and Changes in Medical Decision Making Based on Online Health Information: Path Analysis. *J Med Internet Res* **2020**, *22*, e14095, doi:10.2196/14095.

22. Clifton, J.; Parent, J.; Seehuus, M.; Worrall, G.; Forehand, R.; Domar, A. An Internet-Based Mind/Body Intervention to Mitigate Distress in Women Experiencing Infertility: A Randomized Pilot Trial. *PLoS One* **2020**, *15*, e0229379, doi:10.1371/journal.pone.0229379.

23. Dawson, S.L.; Craig, J.M.; Clarke, G.; Mohebbi, M.; Dawson, P.; Tang, M.L.; Jacka, F.N. Targeting the Infant Gut Microbiota Through a Perinatal Educational Dietary Intervention: Protocol for a Randomized Controlled Trial. *JMIR Res Protoc* **2019**, *8*, e14771, doi:10.2196/14771.

24. Dempsey, A.F.; Wagner, N.; Narwaney, K.; Pyrzanowski, J.; Kwan, B.M.; Kraus, C.; Gleason, K.; Resnicow, K.; Sevick, C.; Cataldi, J.; et al. “Reducing Delays In Vaccination” (REDIVAC) Trial: A Protocol for a Randomised Controlled Trial of a Web-Based, Individually Tailored, Educational Intervention to Improve Timeliness of Infant Vaccination. *BMJ Open* **2019**, *9*, e027968, doi:10.1136/bmjopen-2018-027968.

25. Dennis, C.-L.; Marini, F.; Dick, J.A.; Atkinson, S.; Barrett, J.; Bell, R.; Berard, A.; Berger, H.; Brown, H.K.; Constantin, E.; et al. Protocol for a Randomised Trial Evaluating a Preconception-Early Childhood Telephone-Based Intervention with Tailored e-Health Resources for Women and Their Partners to Optimise Growth and Development among Children in Canada: A Healthy Life Trajectory Initiative (HeLTI Canada). *BMJ Open* **2021**, *11*, e046311, doi:10.1136/bmjopen-2020-046311.

26. Deruelle, P.; Lelorain, S.; Deghilage, S.; Couturier, E.; Guilbert, E.; Berveiller, P.; Sénat, M.V.; Vayssière, C.; Sentilhes, L.; Perrotin, F.; et al. Rationale and Design of EPPOP-ID: A Multicenter Randomized Controlled Trial Using an Electronic-Personalized Program for Obesity in Pregnancy to Improve Delivery. *BMC Pregnancy Childbirth* **2020**, *20*, 602, doi:10.1186/s12884-020-03288-x.

27. Dietrich, J.J.; Benadé, G.L.; Mulaudzi, M.; Kagee, A.; Hornschuh, S.; Makhale, L.M.; Lemos, M.P.; Lazarus, E.; Andrasik, M.P.; Horvath, K.J. “You Are on the Right Track With the App:” Qualitative Analysis of Mobile Phone Use and User Feedback Regarding Mobile Phone Sexual Risk Assessments for HIV Prevention Research. *Front Digit Health* **2021**, *3*, 576514, doi:10.3389/fdgth.2021.576514.

28. Dietrich, J.J.; Otwombe, K.; Pakhomova, T.E.; Horvath, K.J.; Hornschuh, S.; Hlongwane, K.; Closson, K.; Mulaudzi, M.; Smith, P.; Beksinska, M.; et al. High Cellphone Use Associated with Greater Risk of Depression among Young Women Aged 15-24 Years in Soweto and Durban, South Africa. *Glob Health Action* **2021**, *14*, 1936792, doi:10.1080/16549716.2021.1936792.

29. Dietrich, J.J.; Laher, F.; Hornschuh, S.; Nkala, B.; Chimoyi, L.; Otwombe, K.; Kaida, A.; Gray, G.E.; Miller, C. Investigating Sociodemographic Factors and HIV Risk Behaviors Associated With Social Networking Among Adolescents in Soweto, South Africa: A Cross-Sectional Survey. *JMIR Public Health Surveill* **2016**, *2*, e154, doi:10.2196/publichealth.4885.

30. Dowlati, Y.; Ravindran, A.V.; Segal, Z.V.; Stewart, D.E.; Steiner, M.; Meyer, J.H. Selective Dietary Supplementation in Early Postpartum Is Associated with High Resilience against Depressed Mood. *Proc Natl Acad Sci U S A* **2017**, *114*, 3509–3514, doi:10.1073/pnas.1611965114.

31. Downing, K.L.; Best, K.; Campbell, K.J.; Hesketh, K.D. Informing Active Play and Screen Time Behaviour Change Interventions for Low Socioeconomic Position Mothers of Young Children: What Do Mothers Want? *Biomed Res Int* **2016**, *2016*, 2139782, doi:10.1155/2016/2139782.

32. Ekezie, W.; Dallosso, H.; Saravanan, P.; Khunti, K.; Hadjiconstantinou, M. Experiences of Using a Digital Type 2 Diabetes Prevention Application Designed to Support Women with Previous Gestational Diabetes. *BMC Health Serv Res* **2021**, *21*, 772, doi:10.1186/s12913-021-06791-9.

33. El Kazdouh, H.; El-Ammari, A.; Bouftini, S.; El Fakir, S.; El Achhab, Y. Perceptions and Intervention Preferences of Moroccan Adolescents, Parents, and Teachers Regarding Risks and Protective Factors for Risky Sexual Behaviors Leading to Sexually Transmitted Infections in Adolescents: Qualitative Findings. *Reprod Health* **2019**, *16*, 138, doi:10.1186/s12978-019-0801-y.

34. Fonseca, A.; Pereira, M.; Araujo-Pedrosa, A.; Gorayeb, R.; Ramos, M.M.; Canavarro, M.C. Be a Mom: Formative Evaluation of a Web-Based Psychological Intervention to Prevent Postpartum Depression. *Cogn. Behav. Pract.* **2018**, *25*, 473–495, doi:10.1016/j.cbpra.2018.02.002.

35. Forsell, E.; Bendix, M.; Holländare, F.; Szymanska von Schultz, B.; Nasiell, J.; Blomdahl-Wetterholm, M.; Eriksson, C.; Kvarned, S.; Lindau van der Linden, J.; Söderberg, E.; et al. Internet Delivered Cognitive Behavior Therapy for Antenatal Depression: A Randomised Controlled Trial. *J AFFECT DISORD* **2017**, *221*, 56–64, doi:10.1016/j.jad.2017.06.013.

36. GBD 2019 Under-5 Mortality Collaborators Global, Regional, and National Progress towards Sustainable Development Goal 3.2 for Neonatal and Child Health: All-Cause and Cause-Specific Mortality Findings from the Global Burden of Disease Study 2019. *Lancet* **2021**, *398*, 870–905, doi:10.1016/S0140-6736(21)01207-1.

37. Giles, E.L.; Holmes, M.; McColl, E.; Sniehotta, F.F.; Adams, J.M. Acceptability of Financial Incentives for Breastfeeding: Thematic Analysis of Readers’ Comments to UK Online News Reports. *BMC Pregnancy Childbirth* **2015**, *15*, 116, doi:10.1186/s12884-015-0549-5.

38. Govindasamy, D.; Meghij, J.; Kebede Negussi, E.; Clare Baggaley, R.; Ford, N.; Kranzer, K. Interventions to Improve or Facilitate Linkage to or Retention in Pre-ART (HIV) Care and Initiation of ART in Low- and Middle-Income Settings--a Systematic Review. *J Int AIDS Soc* **2014**, *17*, 19032, doi:10.7448/IAS.17.1.19032.

39. Graham, M.L.; Uesugi, K.H.; Niederdeppe, J.; Gay, G.K.; Olson, C.M. The Theory, Development, and Implementation of an e-Intervention to Prevent Excessive Gestational Weight Gain: E-Moms Roc. *Telemed J E Health* **2014**, *20*, 1135–1142, doi:10.1089/tmj.2013.0354.

40. Graham, M.L.; Strawderman, M.S.; Demment, M.; Olson, C.M. Does Usage of an EHealth Intervention Reduce the Risk of Excessive Gestational Weight Gain? Secondary Analysis From a Randomized Controlled Trial. *J Med Internet Res* **2017**, *19*, e6, doi:10.2196/jmir.6644.

41. Granger, D.A.; Hood, K.E.; Dreschel, N.A.; Sergeant, E.; Likos, A. Developmental Effects of Early Immune Stress on Aggressive, Socially Reactive, and Inhibited Behaviors. *Dev. Psychopathol.* **2001**, *13*, 599–610, doi:10.1017/S0954579401003108.

42. Gruver, R.S.; Bishop-Gilyard, C.T.; Lieberman, A.; Gerdes, M.; Virudachalam, S.; Suh, A.W.; Kalra, G.K.; Magge, S.N.; Shults, J.; Schreiner, M.S.; et al. A Social Media Peer Group Intervention for Mothers to Prevent Obesity and Promote Healthy Growth from Infancy: Development and Pilot Trial. *JMIR Res Protoc* **2016**, *5*, e159, doi:10.2196/resprot.5276.

43. Guagliano, J.M.; Armitage, S.M.; Brown, H.E.; Coombes, E.; Fusco, F.; Hughes, C.; Jones, A.P.; Morton, K.L.; van Sluijs, E.M.F. A Whole Family-Based Physical Activity Promotion Intervention: Findings from the Families Reporting Every Step to Health (FRESH) Pilot Randomised Controlled Trial. *Int J Behav Nutr Phys Act* **2020**, *17*, 120, doi:10.1186/s12966-020-01025-3.

44. Guagliano, J.M.; Brown, H.E.; Coombes, E.; Haines, E.S.; Hughes, C.; Jones, A.P.; Morton, K.L.; van Sluijs, E. Whole Family-Based Physical Activity Promotion Intervention: The Families Reporting Every Step to Health Pilot Randomised Controlled Trial Protocol. *BMJ Open* **2019**, *9*, e030902, doi:10.1136/bmjopen-2019-030902.

45. Haemmerli, K.; Znoj, H.; Berger, T. Internet-Based Support for Infertile Patients: A Randomized Controlled Study. *J BEHAV MED* **2010**, *33*, 135–146, doi:10.1007/s10865-009-9243-2.

46. Halili, L.; Liu, R.H.; Weeks, A.; Deonandan, R.; Adamo, K.B. High Maternal Self-Efficacy Is Associated with Meeting Institute of Medicine Gestational Weight Gain Recommendations. *PLoS One* **2019**, *14*, e0226301, doi:10.1371/journal.pone.0226301.

47. Hantsoo, L.; Criniti, S.; Khan, A.; Moseley, M.; Kincler, N.; Faherty, L.J.; Epperson, C.N.; Bennett, I.M. A Mobile Application for Monitoring and Management of Depressed Mood in a Vulnerable Pregnant Population. *Psychiatr Serv* **2018**, *69*, 104–107, doi:10.1176/appi.ps.201600582.

48. Haßdenteufel, K.; Feißt, M.; Brusniak, K.; Lingenfelder, K.; Matthies, L.M.; Wallwiener, M.; Wallwiener, S. Reduction in Physical Activity Significantly Increases Depression and Anxiety in the Perinatal Period: A Longitudinal Study Based on a Self-Report Digital Assessment Tool. *Arch Gynecol Obstet* **2020**, *302*, 53–64, doi:10.1007/s00404-020-05570-x.

49. Heinonen, E.; Blennow, M.; Blomdahl-Wetterholm, M.; Hovstadius, M.; Nasiell, J.; Pohanka, A.; Gustafsson, L.L.; Wide, K. Sertraline Concentrations in Pregnant Women Are Steady and the Drug Transfer to Their Infants Is Low. *Eur J Clin Pharmacol* **2021**, *77*, 1323–1331, doi:10.1007/s00228-021-03122-z.

50. Heinonen, E.; Szymanska-von Schultz, B.; Kaldo, V.; Nasiell, J.; Andersson, E.; Bergmark, M.; Blomdahl-Wetterholm, M.; Forsberg, L.; Forsell, E.; Forsgren, A.; et al. MAGDALENA: Study Protocol of a Randomised, Placebo-Controlled Trial on Cognitive Development at 2 Years of Age in Children Exposed to SSRI in Utero. *BMJ Open* **2018**, *8*, e023281, doi:10.1136/bmjopen-2018-023281.

51. Heuer, L.S.; Rose, M.; Ashwood, P.; Van de Water, J. Decreased Levels of Total Immunoglobulin in Children with Autism Are Not a Result of B Cell Dysfunction. *J. Neuroimmunol.* **2012**, *251*, 94–102, doi:10.1016/j.jneuroim.2012.07.001.

52. Howe, L.D.; Ellison-Loschmann, L.; Pearce, N.; Douwes, J.; Jeffreys, M.; Firestone, R. Ethnic Differences in Risk Factors for Obesity in New Zealand Infants. *J Epidemiol Community Health* **2015**, *69*, 516–522, doi:10.1136/jech-2014-204464.

53. Huang, R.-C.; Silva, D.; Beilin, L.; Neppe, C.; Mackie, K.E.; Roffey, E.; Gibson, L.Y.; D’Vaz, N.; Christian, H.; Reid, C.M.; et al. Feasibility of Conducting an Early Pregnancy Diet and Lifestyle E-Health Intervention: The Pregnancy Lifestyle Activity Nutrition (PLAN) Project. *J Dev Orig Health Dis* **2020**, *11*, 58–70, doi:10.1017/S2040174419000400.

54. Hussain, T.; Smith, P.; Yee, L.M. Mobile Phone-Based Behavioral Interventions in Pregnancy to Promote Maternal and Fetal Health in High-Income Countries: Systematic Review. *JMIR Mhealth Uhealth* **2020**, *8*, e15111, doi:10.2196/15111.

55. Hussain-Shamsy, N.; Shah, A.; Vigod, S.N.; Zaheer, J.; Seto, E. Mobile Health for Perinatal Depression and Anxiety: Scoping Review. *J Med Internet Res* **2020**, *22*, e17011, doi:10.2196/17011.

56. Hutchesson, M.J.; Taylor, R.; Shrewsbury, V.A.; Vincze, L.; Campbell, L.E.; Callister, R.; Park, F.; Schumacher, T.L.; Collins, C.E. Be Healthe for Your Heart: A Pilot Randomized Controlled Trial Evaluating a Web-Based Behavioral Intervention to Improve the Cardiovascular Health of Women with a History of Preeclampsia. *Int J Environ Res Public Health* **2020**, *17*, E5779, doi:10.3390/ijerph17165779.

57. Irving, J.A.; Farb, N.A.S.; Segal, Z.V. Mindfulness-Based Cognitive Therapy for Chronic Depression. In *Handbook of mindfulness: Theory, research, and practice.*; Brown, K.W., Creswell, J.D., Ryan, R.M., Eds.; The Guilford Press: New York, NY, 2015; pp. 348–366 ISBN 978-1-4625-1890-6.

58. James, D.C.; Harville, C.; Sears, C.; Efunbumi, O.; Bondoc, I. Participation of African Americans in E-Health and m-Health Studies: A Systematic Review. *Telemed J E Health* **2017**, *23*, 351–364, doi:10.1089/tmj.2016.0067.

59. Jiskoot, G.; Benneheij, S.H.; Beerthuizen, A.; de Niet, J.E.; de Klerk, C.; Timman, R.; Busschbach, J.J.; Laven, J.S.E. A Three-Component Cognitive Behavioural Lifestyle Program for Preconceptional Weight-Loss in Women with Polycystic Ovary Syndrome (PCOS): A Protocol for a Randomized Controlled Trial. *Reprod Health* **2017**, *14*, 34, doi:10.1186/s12978-017-0295-4.

60. Kamioka, H.; Tsutani, K.; Yamada, M.; Park, H.; Okuizumi, H.; Tsuruoka, K.; Honda, T.; Okada, S.; Park, S.-J.; Kitayuguchi, J.; et al. Effectiveness of Music Therapy: A Summary of Systematic Reviews Based on Randomized Controlled Trials of Music Interventions. *Patient Prefer Adherence* **2014**, *8*, 727–754, doi:10.2147/PPA.S61340.

61. Kandasamy, S.; Anglin, R.; Gaind, L.; Desai, D.; Wahi, G.; Gupta, M.; Anand, S.S. A Qualitative Investigation of Optimal Perinatal Health: The Perspectives of South Asian Grandmothers Living in Southern Ontario, Canada. *BMC Pregnancy Childbirth* **2020**, *20*, 113, doi:10.1186/s12884-020-2762-0.

62. Kelman, A.R.; Evare, B.S.; Barrera, A.Z.; Muñoz, R.F.; Gilbert, P. A Proof‐of‐concept Pilot Randomized Comparative Trial of Brief Internet‐based Compassionate Mind Training and Cognitive‐behavioral Therapy for Perinatal and Intending to Become Pregnant Women. *Clinical Psychology & Psychotherapy* **2018**, *25*, 608–619, doi:10.1002/cpp.2185.

63. Kelman, A.R.; Stanley, M.L.; Barrera, A.Z.; Cree, M.; Heineberg, Y.; Gilbert, P. Comparing Brief Internet-Based Compassionate Mind Training and Cognitive Behavioral Therapy for Perinatal Women: Study Protocol for a Randomized Controlled Trial. *JMIR RES. Protoc.* **2016**, *5*, e65, doi:10.2196/resprot.5332.

64. Kersting, A.; Dölemeyer, R.; Steinig, J.; Walter, F.; Kroker, K.; Baust, K.; Wagner, B. Brief Internet-Based Intervention Reduces Posttraumatic Stress and Prolonged Grief in Parents after the Loss of a Child during Pregnancy: A Randomized Controlled Trial. *PSYCHOTHER PSYCHOSOM* **2013**, *82*, 372–381, doi:10.1159/000348713.

65. Kim, C.; Draska, M.; Hess, M.L.; Wilson, E.J.; Richardson, C.R. A Web-Based Pedometer Programme in Women with a Recent History of Gestational Diabetes. *Diabet Med* **2012**, *29*, 278–283, doi:10.1111/j.1464-5491.2011.03415.x.

66. Kim, H.K.; Niederdeppe, J.; Guillory, J.; Graham, M.; Olson, C.; Gay, G. Determinants of Pregnant Women’s Online Self-Regulatory Activities for Appropriate Gestational Weight Gain. *Health Commun* **2015**, *30*, 922–932, doi:10.1080/10410236.2014.905900.

67. Knowlden, A.P.; Conrad, E. Two-Year Outcomes of the Enabling Mothers to Prevent Pediatric Obesity Through Web-Based Education and Reciprocal Determinism (EMPOWER) Randomized Control Trial. *Health Educ Behav* **2018**, *45*, 262–276, doi:10.1177/1090198117732604.

68. Knowlden, A.; Sharma, M. A Feasibility and Efficacy Randomized Controlled Trial of an Online Preventative Program for Childhood Obesity: Protocol for the EMPOWER Intervention. *JMIR Res Protoc* **2012**, *1*, e5, doi:10.2196/resprot.2141.

69. Krusche, A.; Dymond, M.; Murphy, S.E.; Crane, C. Mindfulness for Pregnancy: A Randomised Controlled Study of Online Mindfulness during Pregnancy. *Midwifery* **2018**, *65*, 51–57, doi:10.1016/j.midw.2018.07.005.

70. Lange, S.; Probst, C.; Gmel, G.; Rehm, J.; Burd, L.; Popova, S. Global Prevalence of Fetal Alcohol Spectrum Disorder Among Children and Youth: A Systematic Review and Meta-Analysis. *JAMA Pediatr* **2017**, *171*, 948–956, doi:10.1001/jamapediatrics.2017.1919.

71. Lau, Y.; Cheng, J.-Y.; Wong, S.-H.; Yen, K.-Y.; Cheng, L.-J. Effectiveness of Digital Psychotherapeutic Intervention among Perinatal Women: A Systematic Review and Meta-Analysis of Randomized Controlled Trials. *World J. Psychiatr.* **2021**, *11*, 133–152, doi:10.5498/wjp.v11.i4.133.

72. Laws, R.A.; Denney-Wilson, E.A.; Taki, S.; Russell, C.G.; Zheng, M.; Litterbach, E.-K.; Ong, K.-L.; Lymer, S.J.; Elliott, R.; Campbell, K.J. Key Lessons and Impact of the Growing Healthy MHealth Program on Milk Feeding, Timing of Introduction of Solids, and Infant Growth: Quasi-Experimental Study. *JMIR Mhealth Uhealth* **2018**, *6*, e78, doi:10.2196/mhealth.9040.

73. Lee, E.W.; Denison, F.C.; Hor, K.; Reynolds, R.M. Web-Based Interventions for Prevention and Treatment of Perinatal Mood Disorders: A Systematic Review. *BMC Pregnancy Childbirth* **2016**, *16*, 38, doi:10.1186/s12884-016-0831-1.

74. Lim, K.; Chan, S.-Y.; Lim, S.L.; Tai, B.C.; Tsai, C.; Wong, S.R.; Ang, S.M.; Yew, T.W.; Tai, E.S.; Yong, E.L. A Smartphone App to Restore Optimal Weight (SPAROW) in Women With Recent Gestational Diabetes Mellitus: Randomized Controlled Trial. *JMIR Mhealth Uhealth* **2021**, *9*, e22147, doi:10.2196/22147.

75. Lindsay, A.C.; Le, Q.; Nogueira, D.L.; Machado, M.M.; Greaney, M.L. Sources of Information about Gestational Weight Gain, Diet and Exercise among Brazilian Immigrant Women Living in the USA: A Cross-Sectional Study. *Public Health Nutr* **2021**, *24*, 5720–5729, doi:10.1017/S1368980021001798.

76. Lindsay, A.C.; Moura Arruda, C.A.; Tavares Machado, M.M.; De Andrade, G.P.; Greaney, M.L. Exploring How Brazilian Immigrant Mothers Living in the USA Obtain Information about Physical Activity and Screen Time for Their Preschool-Aged Children: A Qualitative Study. *BMJ Open* **2018**, *8*, e021844, doi:10.1136/bmjopen-2018-021844.

77. Liu, Z.; Sun, Y.-Y.; Zhong, B.-L. Mindfulness-Based Stress Reduction for Family Carers of People with Dementia. *Cochrane Database Syst Rev* **2018**, *8*, CD012791, doi:10.1002/14651858.CD012791.pub2.

78. Maertens, J.A.; Jimenez-Zambrano, A.M.; Albright, K.; Dempsey, A.F. Using Community Engagement to Develop a Web-Based Intervention for Latinos about the HPV Vaccine. *J Health Commun* **2017**, *22*, 285–293, doi:10.1080/10810730.2016.1275890.

79. Maharjan, S.M.; Poudyal, A.; van Heerden, A.; Byanjankar, P.; Thapa, A.; Islam, C.; Kohrt, B.A.; Hagaman, A. Passive Sensing on Mobile Devices to Improve Mental Health Services with Adolescent and Young Mothers in Low-Resource Settings: The Role of Families in Feasibility and Acceptability. *BMC Med Inform Decis Mak* **2021**, *21*, 117, doi:10.1186/s12911-021-01473-2.

80. Martin, S.L.; McCann, J.K.; Gascoigne, E.; Allotey, D.; Fundira, D.; Dickin, K.L. Engaging Family Members in Maternal, Infant and Young Child Nutrition Activities in Low- and Middle-Income Countries: A Systematic Scoping Review. *Matern Child Nutr* **2021**, *17 Suppl 1*, e13158, doi:10.1111/mcn.13158.

81. Matelski, L.; Van de Water, J. Risk Factors in Autism: Thinking Outside the Brain. *J. Autoimmun.* **2016**, *67*, 1–7, doi:10.1016/j.jaut.2015.11.003.

82. Materia, F.T.; Smyth, J.M.; Heron, K.E.; Hillemeier, M.; Feinberg, M.E.; Fonzi, P.; Symons Downs, D. Preconceptional Health Behavior Change in Women with Overweight and Obesity: Prototype for SMART Strong Healthy Women Intervention. *Mhealth* **2018**, *4*, 24, doi:10.21037/mhealth.2018.06.06.

83. Medical Advisory Secretariat Continuous Subcutaneous Insulin Infusion (CSII) Pumps for Type 1 and Type 2 Adult Diabetic Populations: An Evidence-Based Analysis. *Ont Health Technol Assess Ser* **2009**, *9*, 1–58.

84. Mertens, L.; Braeken, M.A.K.A.; Bogaerts, A. Effect of Lifestyle Coaching Including Telemonitoring and Telecoaching on Gestational Weight Gain and Postnatal Weight Loss: A Systematic Review. *Telemed J E Health* **2019**, *25*, 889–901, doi:10.1089/tmj.2018.0139.

85. Militello, L.K.; Hanna, N.; Nigg, C.R. Pokémon GO Within the Context of Family Health: Retrospective Study. *JMIR Pediatr Parent* **2018**, *1*, e10679, doi:10.2196/10679.

86. Mosdøl, A.; Lidal, I.B.; Straumann, G.H.; Vist, G.E. Targeted Mass Media Interventions Promoting Healthy Behaviours to Reduce Risk of Non-Communicable Diseases in Adult, Ethnic Minorities. *Cochrane Database Syst Rev* **2017**, *2*, CD011683, doi:10.1002/14651858.CD011683.pub2.

87. Nair, U.; Armfield, N.R.; Chatfield, M.D.; Edirippulige, S. The Effectiveness of Telemedicine Interventions to Address Maternal Depression: A Systematic Review and Meta-Analysis. *J Telemed Telecare* **2018**, *24*, 639–650, doi:10.1177/1357633X18794332.

88. Ngai, F.W.; Chan, P.S. A Qualitative Evaluation of Telephone-Based Cognitive-Behavioral Therapy for Postpartum Mothers. *CLIN NURS RES* **2019**, *28*, 852–868, doi:10.1177/1054773818768011.

89. Ngai, F.-W.; Wong, P.; Chung, K.-F.; Leung, K.-Y. The Effect of a Telephone-Based Cognitive Behavioral Therapy on Quality of Life: A Randomized Controlled Trial. *ARCH WOMENS MENT HEALTH* **2017**, *20*, 421–426, doi:10.1007/s00737-017-0722-0.

90. O’Connor, E.; Senger, C.A.; Henninger, M.; Gaynes, B.N.; Coppola, E.; Soulsby Weyrich, M. *Interventions to Prevent Perinatal Depression: A Systematic Evidence Review for the U.S. Preventive Services Task Force*; U.S. Preventive Services Task Force Evidence Syntheses, formerly Systematic Evidence Reviews; Agency for Healthcare Research and Quality (US): Rockville (MD), 2019;

91. Okoth, C.; Opanga, S.; Okalebo, F.; Oluka, M.; Baker Kurdi, A.; Godman, B. Point Prevalence Survey of Antibiotic Use and Resistance at a Referral Hospital in Kenya: Findings and Implications. *Hosp Pract (1995)* **2018**, *46*, 128–136, doi:10.1080/21548331.2018.1464872.

92. Olson, C.M.; Groth, S.W.; Graham, M.L.; Reschke, J.E.; Strawderman, M.S.; Fernandez, I.D. The Effectiveness of an Online Intervention in Preventing Excessive Gestational Weight Gain: The e-Moms Roc Randomized Controlled Trial. *BMC Pregnancy Childbirth* **2018**, *18*, 148, doi:10.1186/s12884-018-1767-4.

93. Østbye, T.; Krause, K.M.; Lovelady, C.A.; Morey, M.C.; Bastian, L.A.; Peterson, B.L.; Swamy, G.K.; Brouwer, R.J.N.; McBride, C.M. Active Mothers Postpartum: A Randomized Controlled Weight-Loss Intervention Trial. *Am J Prev Med* **2009**, *37*, 173–180, doi:10.1016/j.amepre.2009.05.016.

94. Øverby, N.C.; Medin, A.C.; Valen, E.L.; Salvesen, L.; Wills, A.K.; Engeset, D.; Vik, F.N.; Hillesund, E.R. Effectiveness of a Digital Dietary Intervention Program Targeting Young Adults before Parenthood: Protocol for the PREPARED Randomised Controlled Trial. *BMJ Open* **2021**, *11*, e055116, doi:10.1136/bmjopen-2021-055116.

95. Pearson, N.; Ball, K.; Crawford, D. Predictors of Changes in Adolescents’ Consumption of Fruits, Vegetables and Energy-Dense Snacks. *Br J Nutr* **2011**, *105*, 795–803, doi:10.1017/S0007114510004290.

96. Phelan, S.; Brannen, A.; Erickson, K.; Diamond, M.; Schaffner, A.; Muñoz-Christian, K.; Stewart, A.; Sanchez, T.; Rodriguez, V.C.; Ramos, D.I.; et al. “Fit Moms/Mamás Activas” Internet-Based Weight Control Program with Group Support to Reduce Postpartum Weight Retention in Low-Income Women: Study Protocol for a Randomized Controlled Trial. *Trials* **2015**, *16*, 59, doi:10.1186/s13063-015-0573-9.

97. Phelan, S.; Hagobian, T.; Brannen, A.; Hatley, K.E.; Schaffner, A.; Muñoz-Christian, K.; Tate, D.F. Effect of an Internet-Based Program on Weight Loss for Low-Income Postpartum Women: A Randomized Clinical Trial. *JAMA* **2017**, *317*, 2381–2391, doi:10.1001/jama.2017.7119.

98. Poston, L.; Bell, R.; Croker, H.; Flynn, A.C.; Godfrey, K.M.; Goff, L.; Hayes, L.; Khazaezadeh, N.; Nelson, S.M.; Oteng-Ntim, E.; et al. Effect of a Behavioural Intervention in Obese Pregnant Women (the UPBEAT Study): A Multicentre, Randomised Controlled Trial. *Lancet Diabetes Endocrinol* **2015**, *3*, 767–777, doi:10.1016/S2213-8587(15)00227-2.

99. Poudyal, A.; van Heerden, A.; Hagaman, A.; Maharjan, S.M.; Byanjankar, P.; Subba, P.; Kohrt, B.A. Wearable Digital Sensors to Identify Risks of Postpartum Depression and Personalize Psychological Treatment for Adolescent Mothers: Protocol for a Mixed Methods Exploratory Study in Rural Nepal. *JMIR Res Protoc* **2019**, *8*, e14734, doi:10.2196/14734.

100. Power, J.M.; Phelan, S.; Hatley, K.; Brannen, A.; Muñoz-Christian, K.; Legato, M.; Tate, D.F. Engagement and Weight Loss in a Web and Mobile Program for Low-Income Postpartum Women: Fit Moms/Mamás Activas. *Health Educ Behav* **2019**, *46*, 114–123, doi:10.1177/1090198119873915.

101. Recher, M.; Garabedian, C.; Aubry, E.; Sharma, D.; Butruille, L.; Storme, L.; De Jonckheere, J. Opioid Effect on the Autonomic Nervous System in a Fetal Sheep Model. *Arch Gynecol Obstet* **2021**, *304*, 73–80, doi:10.1007/s00404-020-05917-4.

102. Rhodes, A.; Kheireddine, S.; Smith, A.D. Experiences, Attitudes, and Needs of Users of a Pregnancy and Parenting App (Baby Buddy) During the COVID-19 Pandemic: Mixed Methods Study. *JMIR Mhealth Uhealth* **2020**, *8*, e23157, doi:10.2196/23157.

103. Rhodes, A.; Smith, A.D.; Llewellyn, C.H.; Croker, H. Investigating Partner Involvement in Pregnancy and Identifying Barriers and Facilitators to Participating as a Couple in a Digital Healthy Eating and Physical Activity Intervention. *BMC Pregnancy Childbirth* **2021**, *21*, 450, doi:10.1186/s12884-021-03917-z.

104. Rosman, L.; Salmoirago-Blotcher, E.; Cahill, J.; Wuensch, K.L.; Sears, S.F. Depression and Health Behaviors in Women with Peripartum Cardiomyopathy. *Heart Lung* **2017**, *46*, 363–368, doi:10.1016/j.hrtlng.2017.05.004.

105. Russell, C.G.; Denney-Wilson, E.; Laws, R.A.; Abbott, G.; Zheng, M.; Lymer, S.J.; Taki, S.; Litterbach, E.-K.V.; Ong, K.-L.; Campbell, K.J. Impact of the Growing Healthy MHealth Program on Maternal Feeding Practices, Infant Food Preferences, and Satiety Responsiveness: Quasi-Experimental Study. *JMIR Mhealth Uhealth* **2018**, *6*, e77, doi:10.2196/mhealth.9303.

106. Sanabria-Martínez, G.; García-Hermoso, A.; Poyatos-León, R.; González-García, A.; Sánchez-López, M.; Martínez-Vizcaíno, V. Effects of Exercise-Based Interventions on Neonatal Outcomes: A Meta-Analysis of Randomized Controlled Trials. *Am J Health Promot* **2016**, *30*, 214–223, doi:10.1177/0890117116639569.

107. Sandborg, J.; Söderström, E.; Henriksson, P.; Bendtsen, M.; Henström, M.; Leppänen, M.H.; Maddison, R.; Migueles, J.H.; Blomberg, M.; Löf, M. Effectiveness of a Smartphone App to Promote Healthy Weight Gain, Diet, and Physical Activity During Pregnancy (HealthyMoms): Randomized Controlled Trial. *JMIR Mhealth Uhealth* **2021**, *9*, e26091, doi:10.2196/26091.

108. Scherer, S.; Urech, C.; Hösli, I.; Tschudin, S.; Gaab, J.; Berger, T.; Alder, J. Internet-Based Stress Management for Women with Preterm Labour--a Case-Based Experience Report. *Arch Womens Ment Health* **2014**, *17*, 593–600, doi:10.1007/s00737-014-0454-3.

109. Sil, S.; Lynch-Jordan, A.; Ting, T.V.; Peugh, J.; Noll, J.; Kashikar-Zuck, S. Influence of Family Environment on Long-Term Psychosocial Functioning of Adolescents with Juvenile Fibromyalgia. *Arthritis Care Res (Hoboken)* **2013**, *65*, 903–909, doi:10.1002/acr.21921.

110. Singla, D.R.; Lawson, A.; Kohrt, B.A.; Jung, J.W.; Meng, Z.; Ratjen, C.; Zahedi, N.; Dennis, C.-L.; Patel, V. Implementation and Effectiveness of Nonspecialist-Delivered Interventions for Perinatal Mental Health in High-Income Countries A Systematic Review and Meta-Analysis. *JAMA Psychiatry* **2021**, *78*, 498–509, doi:10.1001/jamapsychiatry.2020.4556.

111. Skau, J.K.H.; Nordin, A.B.A.; Cheah, J.C.H.; Ali, R.; Zainal, R.; Aris, T.; Ali, Z.M.; Matzen, P.; Biesma, R.; Aagaard-Hansen, J.; et al. A Complex Behavioural Change Intervention to Reduce the Risk of Diabetes and Prediabetes in the Pre-Conception Period in Malaysia: Study Protocol for a Randomised Controlled Trial. *Trials* **2016**, *17*, 215, doi:10.1186/s13063-016-1345-x.

112. Slocum, S.E.; Wichman, C.L.; Kuehn, S.; Doering, J. Differences in Utilization of Perinatal Psychiatric Teleconsultation Line Between Primary Care and Mental Health Providers. *WMJ* **2021**, *120*, 281–285.

113. Spates, C.R.; Padalino, R.; Hale, A.C.; Germain, C.S.; Nimmo, K.; Kohler, R. A Review of Web-Based Technology in Behavioural Activation. *Clinical Psychologist* **2016**, *20*, 27–35, doi:10.1111/cp.12087.

114. Steegers-Theunissen, R.; Hoek, A.; Groen, H.; Bos, A.; van den Dool, G.; Schoonenberg, M.; Smeenk, J.; Creutzberg, E.; Vecht, L.; Starmans, L.; et al. Pre-Conception Interventions for Subfertile Couples Undergoing Assisted Reproductive Technology Treatment: Modeling Analysis. *JMIR Mhealth Uhealth* **2020**, *8*, e19570, doi:10.2196/19570.

115. Stremler, R.; Hodnett, E.; Kenton, L.; Lee, K.; Weiss, S.; Weston, J.; Willan, A. Effect of Behavioural-Educational Intervention on Sleep for Primiparous Women and Their Infants in Early Postpartum: Multisite Randomised Controlled Trial. *BMJ* **2013**, *346*, f1164, doi:10.1136/bmj.f1164.

116. Taylor, R.; Shrewsbury, V.A.; Vincze, L.; Campbell, L.; Callister, R.; Park, F.; Schumacher, T.; Collins, C.; Hutchesson, M. Be Healthe for Your Heart: Protocol for a Pilot Randomized Controlled Trial Evaluating a Web-Based Behavioral Intervention to Improve the Cardiovascular Health of Women With a History of Preeclampsia. *Front Cardiovasc Med* **2019**, *6*, 144, doi:10.3389/fcvm.2019.00144.

117. Thompson, E.L.; Vamos, C.A.; Daley, E.M. Physical Activity during Pregnancy and the Role of Theory in Promoting Positive Behavior Change: A Systematic Review. *J Sport Health Sci* **2017**, *6*, 198–206, doi:10.1016/j.jshs.2015.08.001.

118. Thorsell, A.; Nätt, D. Maternal Stress and Diet May Influence Affective Behavior and Stress-Response in Offspring via Epigenetic Regulation of Central Peptidergic Function. *Environ Epigenet* **2016**, *2*, dvw012, doi:10.1093/eep/dvw012.

119. Turner, K.; Reynolds, J.N.; McGrath, P.; Lingley-Pottie, P.; Huguet, A.; Hewitt, A.; Green, C.; Wozney, L.; Mushquash, C.; Muhajarine, N.; et al. Guided Internet-Based Parent Training for Challenging Behavior in Children With Fetal Alcohol Spectrum Disorder (Strongest Families FASD): Study Protocol for a Randomized Controlled Trial. *JMIR Res Protoc* **2015**, *4*, e112, doi:10.2196/resprot.4723.

120. Uddin, M.F.; Molyneux, S.; Muraya, K.; Hossain, M.A.; Islam, M.A.; Shahid, A.S.M.S.B.; Zakayo, S.M.; Njeru, R.W.; Jemutai, J.; Berkley, J.A.; et al. Gender-Related Influences on Adherence to Advice and Treatment-Seeking Guidance for Infants and Young Children Post-Hospital Discharge in Bangladesh. *Int J Equity Health* **2021**, *20*, 64, doi:10.1186/s12939-021-01404-7.

121. Upadhyay, U.D.; Cockrill, K.; Freedman, L.R. Informing Abortion Counseling: An Examination of Evidence-Based Practices Used in Emotional Care for Other Stigmatized and Sensitive Health Issues. *Patient Educ Couns* **2010**, *81*, 415–421, doi:10.1016/j.pec.2010.08.026.

122. Urech, C.; Scherer, S.; Emmenegger, M.; Gaab, J.; Tschudin, S.; Hoesli, I.; Berger, T.; Alder, J. Efficacy of an Internet-Based Cognitive Behavioral Stress Management Training in Women with Idiopathic Preterm Labor: A Randomized Controlled Intervention Study. *J. Psychosomat. Res.* **2017**, *103*, 140–146, doi:10.1016/j.jpsychores.2017.10.014.

123. van den Heuvel, J.F.M.; Teunis, C.J.; Franx, A.; Crombag, N.M.T.H.; Bekker, M.N. Home-Based Telemonitoring versus Hospital Admission in High Risk Pregnancies: A Qualitative Study on Women’s Experiences. *BMC Pregnancy Childbirth* **2020**, *20*, 77, doi:10.1186/s12884-020-2779-4.

124. van den Heuvel, J.F.; Groenhof, T.K.; Veerbeek, J.H.; van Solinge, W.W.; Lely, A.T.; Franx, A.; Bekker, M.N. EHealth as the Next-Generation Perinatal Care: An Overview of the Literature. *J Med Internet Res* **2018**, *20*, e202, doi:10.2196/jmir.9262.

125. Van der Geest, K.E.; Mérelle, S.Y.M.; Rodenburg, G.; Van de Mheen, D.; Renders, C.M. Cross-Sectional Associations between Maternal Parenting Styles, Physical Activity and Screen Sedentary Time in Children. *BMC Public Health* **2017**, *17*, 753, doi:10.1186/s12889-017-4784-8.

126. van Grieken, A.; Vlasblom, E.; Wang, L.; Beltman, M.; Boere-Boonekamp, M.M.; L’Hoir, M.P.; Raat, H. Personalized Web-Based Advice in Combination With Well-Child Visits to Prevent Overweight in Young Children: Cluster Randomized Controlled Trial. *J Med Internet Res* **2017**, *19*, e268, doi:10.2196/jmir.7115.

127. Varagiannis, P.; Magriplis, E.; Risvas, G.; Vamvouka, K.; Nisianaki, A.; Papageorgiou, A.; Pervanidou, P.; Chrousos, G.P.; Zampelas, A. Effects of Three Different Family-Based Interventions in Overweight and Obese Children: The “4 Your Family” Randomized Controlled Trial. *Nutrients* **2021**, *13*, 341, doi:10.3390/nu13020341.

128. Vaughn, L.M.; Wagner, E.; Jacquez, F. A Review of Community-Based Participatory Research in Child Health. *MCN Am J Matern Child Nurs* **2013**, *38*, 48–53, doi:10.1097/NMC.0b013e31826591a3.

129. Wassef, A.; Wassef, E. Telemedicine in Perinatal Mental Health: Perspectives. *J. Psychosomat. Obstet. Gynecol.*, doi:10.1080/0167482X.2021.2024162.

130. Wenze, S.J.; Miers, Q.A.; Battle, C.L. Postpartum Mental Health Care for Mothers of Multiples: A Qualitative Study of New Mothers’ Treatment Preferences. *J. Psychiatr. Pract.* **2020**, *26*, 201–214, doi:10.1097/PRA.0000000000000469.

131. Westerhoff, B.; Troesken, A.; Renneberg, B. about:blank? Online Interventions for Postpartum Depression. *Verhaltenstherapie* **2019**, *29*, 254–264, doi:10.1159/000496095.

132. Whitaker, K.M.; Hung, P.; Alberg, A.J.; Hair, N.L.; Liu, J. Variations in Health Behaviors among Pregnant Women during the COVID-19 Pandemic. *Midwifery* **2021**, *95*, 102929, doi:10.1016/j.midw.2021.102929.

133. Whitaker, K.M.; Wilcox, S.; Liu, J.; Blair, S.N.; Pate, R.R. Pregnant Women’s Perceptions of Weight Gain, Physical Activity, and Nutrition Using Theory of Planned Behavior Constructs. *J Behav Med* **2016**, *39*, 41–54, doi:10.1007/s10865-015-9672-z.

134. White, B.; Giglia, R.C.; White, J.A.; Dhaliwal, S.; Burns, S.K.; Scott, J.A. Gamifying Breastfeeding for Fathers: Process Evaluation of the Milk Man Mobile App. *JMIR Pediatr Parent* **2019**, *2*, e12157, doi:10.2196/12157.

135. Widman, L.; Nesi, J.; Choukas-Bradley, S.; Prinstein, M.J. Safe Sext: Adolescents’ Use of Technology to Communicate about Sexual Health with Dating Partners. *J Adolesc Health* **2014**, *54*, 612–614, doi:10.1016/j.jadohealth.2013.12.009.

136. Wise, L.A.; Rothman, K.J.; Mikkelsen, E.M.; Sørensen, H.T.; Riis, A.H.; Hatch, E.E. A Prospective Cohort Study of Physical Activity and Time to Pregnancy. *Fertil Steril* **2012**, *97*, 1136-1142.e1-4, doi:10.1016/j.fertnstert.2012.02.025.

137. Wu, J.J.Y.; Ahmad, N.; Samuel, M.; Logan, S.; Mattar, C.N.Z. The Influence of Web-Based Tools on Maternal and Neonatal Outcomes in Pregnant Adolescents or Adolescent Mothers: Mixed Methods Systematic Review. *J Med Internet Res* **2021**, *23*, e26786, doi:10.2196/26786.

138. Xu, T.; He, Y.; Dainelli, L.; Yu, K.; Detzel, P.; Silva-Zolezzi, I.; Volger, S.; Fang, H. Healthcare Interventions for the Prevention and Control of Gestational Diabetes Mellitus in China: A Scoping Review. *BMC Pregnancy Childbirth* **2017**, *17*, 171, doi:10.1186/s12884-017-1353-1.

139. Xue, W.L.; Shorey, S.; Wang, W.; He, H.-G. Fathers’ Involvement during Pregnancy and Childbirth: An Integrative Literature Review. *Midwifery* **2018**, *62*, 135–145, doi:10.1016/j.midw.2018.04.013.

140. Zhao, L.; Chen, J.; Lan, L.; Deng, N.; Liao, Y.; Yue, L.; Chen, I.; Wen, S.W.; Xie, R. Effectiveness of Telehealth Interventions for Women With Postpartum Depression: Systematic Review and Meta-Analysis. *JMIR mHealth uHealth* **2021**, *9*, e32544, doi:10.2196/32544.

141. Tomás, C.C.; Oliveira, E.; Sousa, D.; Uba-Chupel, M.; Furtado, G.; Rocha, C.; Teixeira, A.; Ferreira, P.; Alves, C.; Gisin, S.; et al. Proceedings of the 3rd IPLeiria’s International Health Congress: Leiria, Portugal. 6-7 May 2016. *BMC Health Serv Res* **2016**, *16*, 200, s12913-016-1423–1425, doi:10.1186/s12913-016-1423-5.

142. Chambers, D.; Simpson, L.; Hill-Briggs, F.; Neta, G.; Vinson, C.; Chambers, D.; Beidas, R.; Marcus, S.; Aarons, G.; Hoagwood, K.; et al. Proceedings of the 8th Annual Conference on the Science of Dissemination and Implementation: Washington, DC, USA. 14-15 December 2015. *Implementation Sci* **2016**, *11*, 100, s13012-016-0452–0, doi:10.1186/s13012-016-0452-0.

143. Ashford, M.T.; Olander, E.K.; Rowe, H.; Fisher, J.R.W.; Ayers, S. Feasibility and Acceptability of a Web-Based Treatment with Telephone Support for Postpartum Women With Anxiety: Randomized Controlled Trial. *JMIR Ment. Health* **2018**, *5*, e19, doi:10.2196/mental.9106.

144. Baylis, R.; Ekdahl, J.; Haines, H.; Rubertsson, C. Women’s Experiences of Internet-Delivered Cognitive Behaviour Therapy (ICBT) for Fear of Birth. *Women and Birth* **2020**, *33*, e227–e233, doi:10.1016/j.wombi.2019.05.006.

145. Bijlholt, M.; Ameye, L.; Van Uytsel, H.; Devlieger, R.; Bogaerts, A. The INTER-ACT E-Health Supported Lifestyle Intervention Improves Postpartum Food Intake and Eating Behavior, but Not Physical Activity and Sedentary Behavior—A Randomized Controlled Trial. *Nutrients* **2021**, *13*, 1287, doi:10.3390/nu13041287.

146. Bogaerts, A.; Ameye, L.; Bijlholt, M.; Amuli, K.; Heynickx, D.; Devlieger, R. INTER-ACT: Prevention of Pregnancy Complications through an e-Health Driven Interpregnancy Lifestyle Intervention – Study Protocol of a Multicentre Randomised Controlled Trial. *BMC Pregnancy Childbirth* **2017**, *17*, 154, doi:10.1186/s12884-017-1336-2.

147. Danaher, B.G.; Milgrom, J.; Seeley, J.R.; Stuart, S.; Schembri, C.; Tyler, M.S.; Ericksen, J.; Lester, W.; Gemmill, A.W.; Lewinsohn, P. Web-Based Intervention for Postpartum Depression: Formative Research and Design of the MomMoodBooster Program. *JMIR Res Protoc* **2012**, *1*, e18, doi:10.2196/resprot.2329.

148. Danaher, B.G.; Milgrom, J.; Seeley, J.R.; Stuart, S.; Schembri, C.; Tyler, M.S.; Ericksen, J.; Lester, W.; Gemmill, A.W.; Kosty, D.B.; et al. MomMoodBooster Web-Based Intervention for Postpartum Depression: Feasibility Trial Results. *J Med Internet Res* **2013**, *15*, e242, doi:10.2196/jmir.2876.

149. Drozd, F.; Haga, S.M.; Brendryen, H.; Slinning, K. An Internet-Based Intervention (Mamma Mia) for Postpartum Depression: Mapping the Development from Theory to Practice. *JMIR Res Protoc* **2015**, *4*, e120, doi:10.2196/resprot.4858.

150. Milgrom, J.; Danaher, B.G.; Gemmill, A.W.; Holt, C.; Holt, C.J.; Seeley, J.R.; Tyler, M.S.; Ross, J.; Ericksen, J. Internet Cognitive Behavioral Therapy for Women With Postnatal Depression: A Randomized Controlled Trial of MumMoodBooster. *J Med Internet Res* **2016**, *18*, e54, doi:10.2196/jmir.4993.

151. Ngai, F.W.; Chan, P.S. A Qualitative Evaluation of Telephone-Based Cognitive-Behavioral Therapy for Postpartum Mothers. *Clin Nurs Res* **2019**, *28*, 852–868, doi:10.1177/1054773818768011.

152. O’Shea, A.; Kaplan, K.; Solomon, P.; Salzer, M.S. Randomized Controlled Trial of an Internet-Based Educational Intervention for Mothers With Mental Illnesses: An 18-Month Follow-Up. *PS* **2019**, *70*, 732–735, doi:10.1176/appi.ps.201800391.

153. Sadigursky, A. Move My Mood: Development and Evaluation of a Mobile Mental Health Self-Help App Using Behavioral Activation for Women with Postpartum Depression., Alliant International University, 2018.

154. Shahsavan, F.; Akbari, N.; Gharraee, B.; Abolghasemi, J.; Khedmat, L. The Effect of Internet‐based Guided Self‐help Cognitive‐behavioral Therapies on Iranian Women’s Psychological Symptoms and Preferred Method of Childbirth. *Perspect Psychiatr Care* **2021**, *57*, 138–147, doi:10.1111/ppc.12535.

155. Solness, C.L.; Kroska, E.B.; Holdefer, P.J.; O’Hara, M.W. Treating Postpartum Depression in Rural Veterans Using Internet Delivered CBT: Program Evaluation of MomMoodBooster. *J Behav Med* **2021**, *44*, 454–466, doi:10.1007/s10865-020-00188-5.

156. Ugarriza DN; Schmidt L Telecare for Women with Postpartum Depression. *J PSYCHOSOC NURS MENT HEALTH SERV* **2006**, *44*, 37–47, doi:10.3928/02793695-20060101-08.

157. Beck, A.; Dimidjian, S.; Sherwood, N.; Goodman, S.; Welch, S.; Ludman, E.; Boggs, J.; Metcalf, C.; Simon, G. C4-5: Behavioral Activation Therapy for Perinatal Depression: Preliminary Results from a Multi-Site Randomized Trial. *Clinical Medicine & Research* **2014**, *12*, 103–103, doi:10.3121/cmr.2014.1250.c4-5.

158. Dimidjian, S.; Goodman, S.H.; Sherwood, N.E.; Simon, G.E.; Ludman, E.; Gallop, R.; Welch, S.S.; Boggs, J.M.; Metcalf, C.A.; Hubley, S.; et al. A Pragmatic Randomized Clinical Trial of Behavioral Activation for Depressed Pregnant Women. *Journal of Consulting and Clinical Psychology* **2017**, *85*, 26–36, doi:10.1037/ccp0000151.

159. Forsell, E.; Bendix, M.; Holländare, F.; Szymanska von Schultz, B.; Nasiell, J.; Blomdahl-Wetterholm, M.; Eriksson, C.; Kvarned, S.; Lindau van der Linden, J.; Söderberg, E.; et al. Internet Delivered Cognitive Behavior Therapy for Antenatal Depression: A Randomised Controlled Trial. *J AFFECT DISORD* **2017**, *221*, 56–64, doi:10.1016/j.jad.2017.06.013.

160. Kieffer, E.C.; Caldwell, C.H.; Welmerink, D.B.; Welch, K.B.; Sinco, B.R.; Guzmán, J.R. Effect of the Healthy MOMs Lifestyle Intervention on Reducing Depressive Symptoms Among Pregnant Latinas. *American Journal of Community Psychology* **2013**, *51*, 76–89, doi:10.1007/s10464-012-9523-9.

161. Bagnall, M. Long-Term Follow-Up of NetmumsHWD: A Feasibility Randomised Controlled Trial of Telephone Supported Online Behavioural Activation for Postnatal Depression at 16 Months Post-Randomisation, University of Exeter, 2014.

162. O’Mahen, H.A.; Woodford, J.; McGinley, J.; Warren, F.C.; Richards, D.A.; Lynch, T.R.; Taylor, R.S. Internet-Based Behavioral Activation--Treatment for Postnatal Depression (Netmums): A Randomized Controlled Trial. *J Affect Disord* **2013**, *150*, 814–822, doi:10.1016/j.jad.2013.03.005.

163. O’Mahen, H.A.; Richards, D.A.; Woodford, J.; Wilkinson, E.; McGinley, J.; Taylor, R.S.; Warren, F.C. Netmums: A Phase II Randomized Controlled Trial of a Guided Internet Behavioural Activation Treatment for Postpartum Depression. *Psychol. Med.* **2014**, *44*, 1675–1689, doi:10.1017/S0033291713002092.

164. O’Mahen, H.A.; Wilkinson, E.; Bagnall, K.; Richards, D.A.; Swales, A. Shape of Change in Internet Based Behavioral Activation Treatment for Depression. *Behaviour Research and Therapy* **2017**, *95*, 107–116, doi:10.1016/j.brat.2017.05.011.

165. Obikane, E.; Baba, T.; Shinozaki, T.; Obata, S.; Nakanishi, S.; Murata, C.; Ushio, E.; Suzuki, Y.; Shirakawa, N.; Honda, M.; et al. Internet-Based Behavioural Activation to Improve Depressive Symptoms and Prevent Child Abuse in Postnatal Women (SmartMama): A Protocol for a Pragmatic Randomized Controlled Trial. *BMC Pregnancy Childbirth* **2021**, *21*, 314, doi:10.1186/s12884-021-03767-9.

166. Singla, D.R.; Meltzer-Brody, S.E.; Silver, R.K.; Vigod, S.N.; Kim, J.J.; La Porte, L.M.; Ravitz, P.; Schiller, C.E.; Schoueri-Mychasiw, N.; Hollon, S.D.; et al. Scaling Up Maternal Mental Healthcare by Increasing Access to Treatment (SUMMIT) through Non-Specialist Providers and Telemedicine: A Study Protocol for a Non-Inferiority Randomized Controlled Trial. *Trials* **2021**, *22*, 186, doi:10.1186/s13063-021-05075-1.

167. Singla, D.R.; Hossain, S.; Ravitz, P.; Schiller, C.E.; Andrejek, N.; Kim, J.; La Porte, L.; Meltzer-Brody, S.E.; Silver, R.; Vigod, S.N.; et al. Adapting Behavioral Activation for Perinatal Depression and Anxiety in Response to the COVID-19 Pandemic and Racial Injustice. *J Affect Disord* **2022**, *299*, 180–187, doi:10.1016/j.jad.2021.12.006.
